# Synthesis and Properties of Annulated 2-(Azaar-2-yl)- and 2,2'-Di(azaar-2-yl)-9,9'-spirobifluorenes

**DOI:** 10.3390/molecules181113680

**Published:** 2013-11-05

**Authors:** Jing Lu Liang, Hyochang Cha, Yurngdong Jahng

**Affiliations:** College of Pharmacy, Yeungnam University, Gyeongsan 712-749, Korea

**Keywords:** 9,9'-spirobifluorene, quinoline, 1,8-naphthyridine, benzo[*h*]quinoline, 1,10-phenanthroline, XRD, photoluminescence

## Abstract

A series of 9,9'-spirobifluorene-derived *N*-heterocycles were prepared by the reactions of 8,9-dihydrospiro(benzo[*b*]fluorene-11,9'-fluoren)-6(7*H*)-one and 8,8',9,9'-tetrahydro-11,11'-spirobi(benzo[*b*]fluorene)-6,6'(7*H*,7'*H*)-dione with a series of 2-amino-arenecarbaldehydes such as 2-aminobenzaldehyde, 2-aminonicotinealdehyde, 1-amino-2-naphthaldehyde, and 8-aminoquinoline-7-carbaldehyde. In addition to the absorption maxima based on the parent 9,9'-spirobifluorene skeleton in the 225–234, 239–280, 296–298, and 308–328 nm regions, the absorptions due to the π-π* transitions of the heterocycles were observed in the 351–375 nm region in the UV absorption spectra. All the compounds showed strong photoluminescences in the 390–430 nm region.

## 1. Introduction

9,9'-Spirobifluorene (**1**) as well as its derivatives have been of interest due to their characteristic structures [1], of which the early studies were focused not only on the molecular recognition of various α-aminoalcohols by 9,9'-spirobifluorene-based crown ethers [2,3] and of monosaccharides by dendritic cleft receptors [4,5,6], but also on the inclusion of compounds with hydrocarbons [7], carbohydrates [8,9], and α-hydroxycarboxylates [10]. However, interest in 9,9'-spirobifluorenes, especially 9,9'-spirobifluorene-based oligomers, as well as polymers has recently switched to applications in molecular electronic devices for light emitting devices [11,12,13,14,15,16], hybrid porous solid [17] and catalysis [18].

Nevertheless, only a few of monomeric 2,2'-di(heteroaryl)-9,9'-spirobifluorenes such as 2,2'-di(benzo[*h*]quinol-2-yl)-9,9'-spirobifluorene [19], 2,2'-di(1,10-phenanthrol-2-yl)-9,9'-spiro-bifluorene [20,21], 2,2',7,7'-tetra(benzo[*h*]quinol-2-yl)-9,9'-spirobifluorene [22], and 2,2'-di-(benzo- [*b*]-1,10-phenanthrolin-2-yl)-9,9'-spirobifluorene [23] have been reported, and even fewer monoazaaryl-9,9'-spirobifluorenes, such as 2-(4-phenylquinol-2-yl)-9,9'-spirobifluorene [24] and a series of 2-(azaar-2-yl)-9,9'-spirobifluorenes [25] were reported. Even though some of these showed promising photophysical properties for OLED applications, no systematic approaches for the preparation or for the examination of their properties of monomeric 9,9'-spirobifluorene-based heteroaromatics have been pursued as yet. Our interest in the preparation and properties of 9,9'-spirobifluorene-derived polydentates [23,25] encouraged us to thus study a series of 3,2''-annulated 2-(azaheteroar-2''-yl)-9,9'-spirobifluorenes and 3,2'';3',2''-annulated 2,2'-di(azaheteroar-2''-yl)-9,9'-spirobifluorenes.

## 2. Results and Discussion

### 2.1. Synthesis

**Scheme 1:**
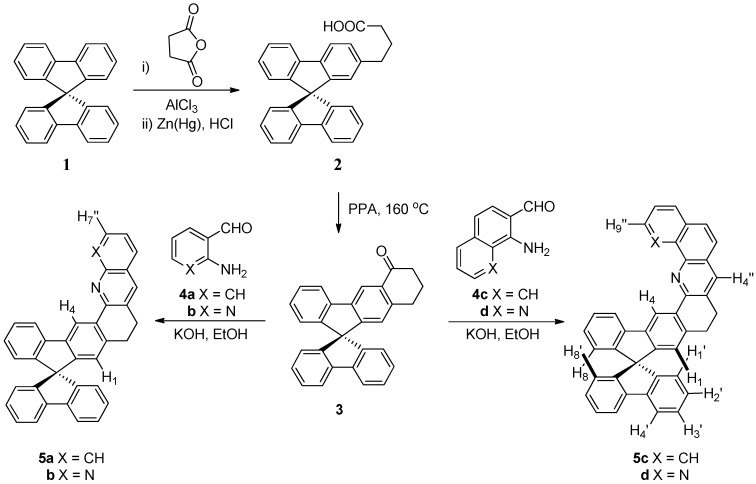
Synthesis of annulated monoazaar-2-yl-9,9'-spirobifluorenes.

**Scheme 2:**
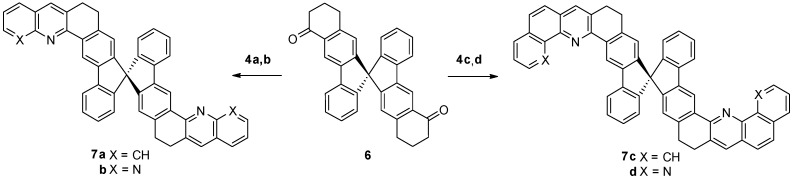
Synthesis of annulated 2,2'-di(azaar-2-yl)-9,9'-spirobifluorenes.

Synthesis of 9,9'-spirobifluorene-derived *N*-heterocycles was straightforward, as shown in Scheme 1 and Scheme 2. Friedländer condensation of **3** with a series of *o*-aminoaldehydes **4** such as 2-aminobenzaldehyde [26], 2-aminonicotinaldehyde [27], 1-aminonaphthalene-2-carbaldehyde, and 8-aminoquinoline-7-carbaldehyde [28], afforded the corresponding heteroaromatics **5** with a 9,9'-spirobifluorene skeleton in 76%–82% yields.

Similarly, the same Friedländer reaction of the diketone **6** [2] with a series of *o*-aminoarenecarbaldehydes afforded the corresponding ligands **7** in 75%–82% yields. The prerequisite ketones **3** and **6** were prepared by polyphosphoric acid (PPA)-catalyzed Friedel-Crafts acylation of known 4-[9,9'-spirobi(fluoren)-7-yl]butanoic acid (**2**) and 4,4'-(9,9'-spirobi[fluorene]-7,7'-diyl)dibutanoic acid [2] in 70% and 78% yields, respectively.

### 2.2. Structural and Thermal Properties

**Figure 1:**
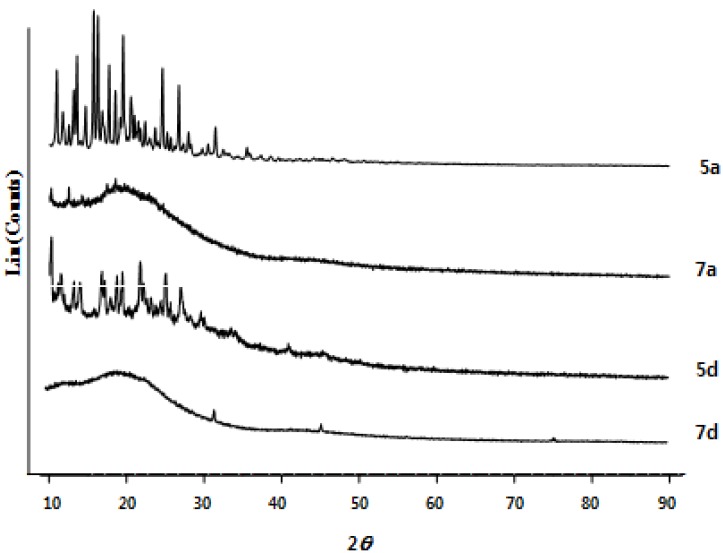
X-ray diffractograms of selected compounds **5a**, **5d**, **7a**, and **7d** in powder state.

The crystallinity of **5** and **7** was accessed by XRD (X-ray diffraction) as shown in Figure 1. All the X-ray diffractograms of the monoheteroaryl systems **5a**,**d**, including **5b**,**c** (not shown) showed numerous distinctive peaks indicating sharp crystalline nature while those of diheteroaryl systems **7a**,**d** showed a characteristic amorphous halo and were free from any type of crystalline peaks.

The thermal behaviors of **5** and **7** were analyzed by differential scanning calorimetry (DSC). All the compounds exhibited a single sharp endothermic peak at the melting points. However, neither glass transition temperatures (Tg) nor crystallization temperature (Tc) were observed for any of the compounds. 

### 2.3. Spectroscopic Properties

2D and DEPT NMR experiments and comparison with the NMR data of the parent 9,9'-spirobifluorene [29] and related compounds reported previously [23,25] allowed us to assign all the proton resonances of the products, which are summarized in Table 1. Some proton resonances were characteristic enough to provide a probe for the suggested structure. The resonances of H4 of the 9,9'-spirobifluorene moiety were generally deshielded by the neighboring *N* in the cavity, and thus resonated in the δ 9.01–9.38 region, showing significant down-field shifts by ∆δ 1.17–1.54 ppm as compared with the parent 9,9'-spirobifluorene. On the other hand, the H1, H8, H1', and H8' protons of the 9,9'-spirobifluorene moiety were upfield-shifted due to the anisotropic effect of the two neighboring phenyl groups, and thus resonated in the δ 6.52–6.87 range. The H1 resonances of **5** and **7** were upfield-shifted by ∆δ 0.05–0.21 ppm, except in **7c**, while those of the H8 protons of **7** are slightly downfield-shifted compared to the corresponding proton resonance of the parent 9,9'-spirobifluorene. Additionally, the protons at the *peri*-position (H7'') of **5a** and **7a** resonated at δ 8.21 and 8.22, respectively, and H10'' of the benzo[*h*]quinoline moiety of **5c** and **7c** resonated at δ 9.62 and 9.63, respectively, as a doublet of doublets (^3^*J* = 7.8 Hz and ^4^*J* = 1.0 Hz), evidencing the anisotropic effect of the neighboring pyridine as well as the electronic influence of the lone pair electrons on the central *N*. In addition, the protons adjacent to nitrogen (H7'' of **5b** and **7b**, H9'' of **5d** and **7d**) give one of the characteristic chemical shifts (δ 9.08 and 9.09 for H7'' and 9.26 and 9.28 for H9'', respectively) as well as relatively small and characteristic ^3^*J* coupling constants (^3^*J* = 4.3 Hz) [30]. Similar characteristic proton resonances for selected protons were also observed in the ^1^H-NMR spectra of **7**.

**Table 1:** Selected proton resonances for compounds **5** and **7**.

	H1 ^a^	H4	H8	H1'	H7''	H8''	H9''	H10''
**1**	7.63	7.84 [29]	-	-	-	-	-	-
**5a**	6.62	9.01	6.72	6.78	-	8.21	-	-
**5b**	6.60	9.25	6.72	6.78	9.08	-	-	-
**5c**	6.52	9.22	6.72	6.78	-	7.72	7.84	9.62
**5d**	6.62	9.34	6.71	6.78	8.25	7.64	9.26	-
**7a**	6.67	9.05	6.79	-	-	8.22	-	-
**7b**	6.69	9.28	6.80	6.69	9.09	-	-	-
**7c**	6.74	9.25	6.87	6.74	8.18	7.73	7.84	9.63
**7d**	6.68	9.38	6.80	-	8.26	7.65	9.28	-

^a^ Numbering pattern and numbering were shown in Scheme 1.

**Figure 2:**
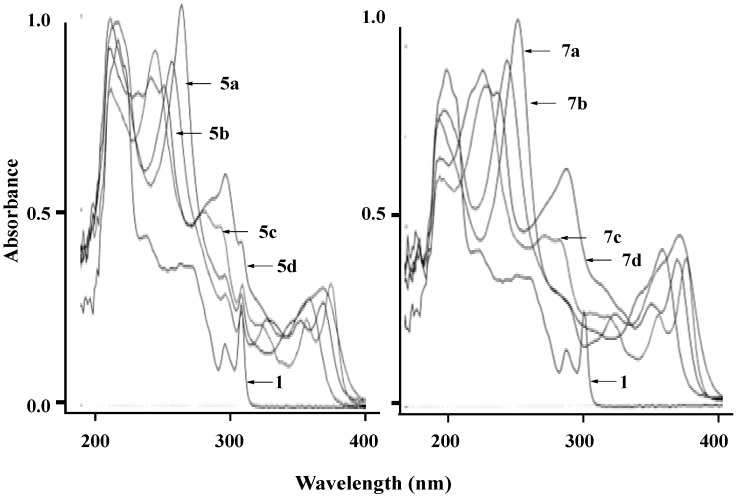
UV absorption spectra of **1**, **5** and **7** in 95% EtOH (1.0 × 10^−5^ M).

UV absorption spectra of 9,9'-spirobifluorene (**1**) and its derivatives **5** and **7** were obtained in 95% EtOH (1.0 × 10^−^^5^ M) at 298 K (Figure 2) and results are summarized in Table 2. Five major absorption maxima due to the π-π* transition were observed in the 225–234, 239–280, 296–298, 308–328, and 351–375 nm regions. The wavelength of the absorption maximum is highly dependent on the nature of the heterocycles attached to the 9,9'-spirobifluorene core. The systems with benzo[*h*]quinoline(s) (compounds **5c** and **7c**), and 1,10-phenanthroline(s) (compounds **5d** and **7d**) showed additional absorptions in the 351–375 nm range, which were not observed in the parent 9,9'-spirobifluorene, indicating that these absorptions were from the π-π* transitions of the heterocycles in the system. Such absorptions in compounds with more aromatic rings such as **5c**,**d** and **7c**,**d** were resolved to show two absorptions in the 351–357 and 359–375 nm regions, respectively, presumably due to the extended delocalization of π-electrons.

**Table 2:** UV absorption and fluorescence spectral data of spirobifluorene, **5** and **7**.

Comp	λ_max_/nm (1 × 10^−5^ M in 95% EtOH, log ε)	λ_em_/nm
**1**	225 (4.65) 239 (4.40) 272 (4.29) 296 (3.92) 308 (4.11)	388 (308)
**5a**	213 (4.97) 265 (5.00) 296 (4.53) 308 (4.46) 343 (sh, 4.28) 357 (4.44)	390 (357)
**5b**	219 (4.99) 258 (4.95) 296 (4.45) 308 (4.38) 326 (4.45) 367 (4.48)	430 (367)
**5c**	214 (4.91) 246 (sh, 4.96) 280 (4.70) 293 (4.66) 309 (4.96) 324 (4.31) 355 (4.35) 373 (4.50)	392 (373)
**5d**	212 (4.96) 234 (4.90) 243 (4.93) 252 (4.91) 297 (4.77) 308 (4.63) 351 (4.34) 367 (4.43)	395 (367)
**7a**	214 (4.90) 265 (5.00) 295 (4.48) 343 (sh, 4.80) 359 (4.63)	395 (359)
**7b**	216 (4.92) 258 (4.98) 293 (sh, 4.48) 328 (4.40) 370 (4.67)	395 (370)
**7c**	214 (4.81) 244 (4.95) 283 (4.67) 315 (4.41) 323 (4.40) 357 (4.40) 374 (4.22)	390 (357)
**7d**	214 (4.84) 242 (4.97) 252 (4.94) 297 (4.82) 352 (4.45) 369 (4.61)	397 (369)

* Number in parenthesis is the excitation wavelength for each compound.

The photoluminescence (PL) of **1**, **5**, and **7** at 298 K was studied in EtOH (1.0 × 10^−^^5^ M) to show strong emissions in a 390–430 nm range of, but no correlation between emission maxima and the nature of the heteroaryl skeletons were observed.

## 3. Experimental

### General

Melting points were determined using a Fischer-Jones melting point apparatus and DSC with tin (mp 232.52 °C) as a standard. IR spectra were obtained using a Perkin-Elmer 1330 spectrophotometer. NMR spectra were obtained using a 250 or 600 MHz Bruker-250 spectrometer for ^1^H-NMR and 62.5 or 150 MHz for ^13^C-NMR, respectively, and are reported as parts per million (ppm) from the internal standard tetramethylsilane (TMS) peak. The two-dimensional and DEPT NMR spectra were recorded using Bruker’s standard pulse program. UV absorption spectra were recorded on a JASCO-V550 spectrophotometer and emission spectra on a Perkin Elmer LS50B luminescence spectrometer. Electrospray ionization (ESI) mass spectrometry (MS) experiments were performed on a LCQ advantage-trap mass spectrometer (Thermo Finnigan, San Jose, CA, USA). Elemental analyses were taken on a Hewlett-Packard Model 185B elemental analyzer. XRD analysis was performed by X-ray Diffractometer (Model: MPD for bulk, PANalytical, Westborough, MA, USA) with nickel-filtered CuK*α* radiatiuon (30 kV, 30 mA) at 2*θ* angles ranging from 10° to 90°, a scan speed of 10°/min and a time constant of 1 s. Thermal behaviors of the compounds were analysed using differential scanning calorimeter (DSC Q00) with 1~2 mg of sample sealed in alumina in the range of 40–385 °C increasing temperature in a rate of 10 °C/min. An empty pan was used as a reference, and the DSC baseline, temperature, and enthalpy was calibrated. A nitrogen flow rate for the each scanning calorimetric run was 50 mL/min. The starting 4-[9,9'-spirobi(fluoren)-7-yl]butanoic acid (**2**) [2], *o*-aminoarenecarbaldehydes [26,27,28], and diketone (**6**) [2] were prepared by employing previously reported methods. Chemicals and solvents were of commercial reagent grade and used without further purification. The abbreviations q, np, bq, and phen were used for quinoline, 1,8-naphthyridine, benzo[*h*]quinoline and 1,10-phenanthroline, respectively.

*8,9-Dihydrospiro(benzo[b]fluorene-11,9'-fluoren)-6(7H)-one* (**3**). A solution of 4-[9,9'-spirobi-(fluoren)-7-yl]butanoic acid [2] (0.30 g, 0.75 mmol) in PPA (60 mL) was heated for 40 min and poured to ice water. The precipitate formed was collected and chromatographed on silica gel eluting with CH_2_Cl_2_ to give pale yellow needles (0.20 g, 70%): m.p. 187–189 °C. ^1^H-NMR (CDCl_3_, 250 MHz) δ 8.50 (s, 1H, H5), 7.84 (m, 3H, H4, H4', H4''), 7.38 (m, 3H, H3, H3', H3''), 7.11 (m, 3H, H2, H2', H2''), 6.72 (m, 3H, H1, H1', H1''), 6.62 (s, H10), 2.74 (t, 2H, *J* = 6.5 Hz), 2.64 (t, 2H, *J* = 6.5 Hz), 2.04 (t, 2H, *J* = 6.5 Hz). ^13^C-NMR (CDCl_3_, 62.5 MHz) δ 200.23, 148.09, 148.94, 148.86, 141.73, 141.67, 141.21, 139.91, 127.97, 127.80, 127.65, 127.62, 127.44, 124.06, 123.97, 123.92, 119.92, 119.85, 119.70, 65.90, 35.07, 33.16, 26.15.

*6',7'-Dihydrospiro(fluorene-9,9'-fluorneo[2,3-c]acridine)* (**5a**). A mixture of ketone **3** (384 mg, 1.00 mmol) and 2-aminobenzaldehyde (**4a**, 133 mg, 1.10 mmol) and in absolute EtOH (20 mL) containing saturated alcoholic KOH (1 mL) was refluxed for 5 h. Upon cooling the reaction mixture, a solid was formed, which was collected as a pure yellow crystalline solid product (354 mg, 80%): mp 313.35 °C. IR (KBr) υ 3056, 1593, 1445, 1429 824 cm^−1^. ^1^H-NMR (CDCl_3_, 600 MHz) δ 9.01 (s, 1H, H4), 8.21 (d, *J* = 8.2 Hz, 1H, H8 of q), 8.05 (d, *J* = 7.5 Hz, 1H, H5 of q), 7.90 (s, 1H, H4 of q), 7.85 (d, 2H, *J* = 7.8 Hz, H4' and H5'), 7.75 (d, 1H, *J* = 8.0 Hz, H5), 7.68 (td, 1H, *J* = 8.2, 1.5 Hz, H7 of q), 7.48 (t, *J* = 7.8, 1.5 Hz, H6 of q), 7.40 (td, 1H, *J* = 8.0, 0.8 Hz, H6), 7.37 (td, 2H, *J* = 7.8, 1.2 Hz, H3' and H6'), 7.14 (td, 1H, *J* = 8.0, 0.8 Hz, H7), 7.12 (td, 2H, *J* = 7.8, 1.2 Hz, H2’ and H7'), 6.78 (d, 2H, *J* = 7.8 Hz, H1' and H8'), 6.72 (d, 1H, *J* = 7.8 Hz, H8), 6.62 (s, 1H, H1), 3.03 (t, 2H, *J* = 6.5 Hz), 2.79 (t, 2H, *J* = 6.5 Hz). ^13^C-NMR (CDCl_3_, 62.5 MHz) δ 153.63, 150.53, 148.92, 148.61, 147.70, 141.80, 141.73, 139.65, 134.76, 133.68, 130.70, 129.36, 127.88 (two C’s), 127.82, 127.75, 127.68, 65.89, 28.78, 28.69. MS (ESI): *m*/*z* = 470 [M+H]^+^, Anal. Calcd for C_36_H_23_N: C, 92.08; H, 4.94; N, 2.98. Found: C, 90.95; H, 4.92; N, 2.90.

*6',7'-Dihydrospiro(fluorene-9,9'-indeno[2',1':6,7]naphtho[1,2-b]-1,8-naphthyridine)* (**5b**). The same procedure described above but with 2-aminonicotinaldehyde (**4b**) afforded the desired product as pale yellow needles (82%): m.p. 339.03 °C. ^1^H-NMR (CDCl_3_, 250 MHz) δ 9.25 (s, 1H, H4), 9.08 (dd, *J* = 4.3, 2.0 Hz, 1H, H7 of np), 8.09 (dd, *J* = 8.3, 1.5 Hz, 1H, H5 of np), 8.04 (d, *J* = 8.5 Hz, 1H, H5), 7.90 (s, H4 of np), 7.86 (d, 2H, *J* = 7.5 Hz, H4' and H5'), 7.44-7.34 (m, 4H, H6, H3', H6', H6 of np), 7.11 (td, 2H, *J* = 7.5, 0.8 Hz, H2' and H7'), 7.10 (dd, 1H, *J* = 7.5, 0.8 Hz, H7), 6.78 (d, 2H, *J* = 7.5 Hz, H1' and H8'), 6.72 (d, 1H, *J* = 7.5 Hz, H8), 6.60 (s, 1H, H1), 3.06 (t, 2H, *J* = 6.7 Hz), 2.81 (t, 2H, *J* = 6.7 Hz). ^13^C-NMR (CDCl_3_, 62.5 MHz) δ 153.62, 150.52, 148.92, 148.61, 147.70, 141.80, 141.73, 141.29, 139.65, 134.58, 133.68, 130.70, 129.36, 128.72, 127.88 (two C’s), 1 27.82, 127.74, 127.68, 126.95, 126.07, 124.23, 123.86, 123.39, 120.57, 120.00, 117.64, 65.89, 28.78, 28.68. MS (ESI): *m*/*z* = 471 [M+H]^+^, Anal. Calcd for C_35_H_2__2_N_2_ Calcd for C_35_H_2__2_N_2_**∙**H_2_O C, 86.04; H, 4.95; N, 5.73. Found: C, 85.59; H, 5.01; N, 5.89.

*8,9-Dihydrospiro(benzo[c]fluoreno*[3,2-h]*acridine-11,9'-fluorene)* (**5c**). The same procedure described above for **5a** but with 1-aminonaphthalene-2-carbaldehyde (**4c**) afforded the desired product as pale yellow crystalline solid (79%): m.p. 303.65 °C. ^1^H-NMR (CDCl_3_, 600 MHz) δ 9.62 (dd, 1H, *J* = 7.8, 1.0 Hz, H10 of bq), 9.22 (s, 1H, H4), 8.14 (d, 1H, *J* = 7.8 Hz, H7 of bq), 7.95 (s, 1H, H4 of bq), 7.93 (d, 1H, *J* = 8.4 Hz, H5), 7.88 (d, 2H, *J* = 7.8 Hz, H4' and H5'), 7.84 (td, 1H, *J* = 7.8, 0.6 Hz, H9 of bq), 7.79 (d, 1H, *J* = 9.0 Hz, H5/H6 of bq), 7.72 (td, 1H, *J* = 7.8, 1.2 Hz, H8 of bq), 7.66 (d, 1H, *J* = 9.0 Hz, H6/H5 of bq), 7.46 (td, *J* = 7.5, 1.0 Hz, 1H, H6), 7.40 (td, *J* = 7.5, 1.0 Hz, 2H, H3' and H6'), 7.14 (m, 3H, H7, H2' and H7'), 6.83 (d, *J* = 7.5 Hz, 2H, H1' and H8'), 6.76 (d, 1H, *J* = 7.5 Hz, H8), 6.52 (s, 1H, H1), 3.09 (t, 2H, *J* = 6.5 Hz), 2.85 (t, 2H, *J* = 6.5 Hz). ^13^C-NMR (CDCl_3_, 62.5 MHz) δ 151.82, 150.21, 148.94, 148.64, 145.22, 141.88, 141.72, 141.17, 139.30, 134.97, 134.12, 133.50, 131.80, 130.95, 127.87 (two C’s), 127.83, 127.74 (two C’s), 127.68, 127.23, 126.84, 125.71, 125.01, 124.49, 124.24, 123.92, 123.41, 120.38, 120.00, 117.42, 65.86, 28.62, 28.41. MS (ESI): *m*/*z* = 520 [M+H^+^], Anal. Calcd for C_40_H_25_N**∙**H_2_O: C, 89.36; H, 5.06; N, 2.61. Found: C, 89.21; H, 4.98; N, 2.64.

*8',9'-Dihydrospiro(fluorene-11,9'-indeno[2',1'':6,7]naphtha[1,2-b]-1,10-phenanthroline)* (**5d**). The same procedure described above for **5a** but with 8-aminoquinoline-7-carbaldehyde (**4d**) afforded the desired product as pale yellow crystalline solids (76%): m.p. 395.05 °C. ^1^H-NMR (CDCl_3_, 250 MHz) δ 9.35 (s, 1H, H4), 9.26 (dd, *J* = 4.3, 1.8 Hz, 1H, H9 of phen), 8.25 (dd, *J* = 8.0, 1.8 Hz, 1H, H7 of phen), 8.17 (d, *J* = 7.5 Hz, 1H, H7 of phen), 8.00 (s, 1H, H4 of phen), 7.85 (d, *J* = 7.5 Hz, 2H, H4' and H5'), 7.74 (AB quartet, H5 and H6 of phen), 7.64 (dd, *J* = 8.0, 4.3 Hz, 1H, H8 of phen), 7.40 (td, *J* = 7.5, 1.0 Hz, 1H, H6), 7.37 (td, *J* = 7.5, 1.0 Hz, 2H, H3' and H6'), 7.12 (td, *J* = 7.5, 1.0 Hz, 2H, H2' and H7'), 7.10 (td, *J* = 7.5, 1.0 Hz, 1H, H7), 6.78 (d, *J* = 7.5 Hz, 2H, H1' and H8'), 6.71 (d, *J* = 7.5 Hz, 1H, H8), 6.62 (s, 1H, H1), 3.10 (dd, *J* = 7.5, 6.3 Hz, 2H), 2.82 (dd, *J* = 7.5, 6.3 Hz, 2H). ^13^C-NMR (CDCl_3_, 62.5 MHz) δ 153.70, 150.51, 150.16, 149.00, 148.50, 146.36, 145.07, 142.16, 141.94, 141.71, 141.33, 139.20, 136.17, 134.65, 134.34, 132.66, 128.62, 128.13, 127.86 (two C’s), 127.70 (two C’s), 127.54, 126.29, 125.94, 124.26, 123.69, 123.11, 122.59, 121.04, 119.95, 118.83, 65.89, 28.68, 28.48. MS (ESI): *m*/*z* = 521 [M+H]^+^, Anal. Calcd for C_39_H_25_N_2_**∙**0.75H_2_O: C, 87.70; H, 4.81; N, 5.24. Found C, 87.78; H, 4.81; N, 5.53.

*6,6',7,7'-Tetrahydro-9,9'-spirobi(fluoreno[2,3-c]acridine)* (**7a**). A mixture of ketone **5** (226 mg, 0.50 mmol) and 2-aminobenzaldehyde (**4a**, 133 mg, 2.20 mmol) in absolute EtOH (20 mL) containing saturated alcoholic KOH (1 mL) was refluxed for 5 h. Upon cooling the reaction mixture, the solid was formed, which was collected as a pure pale yellow crystalline solid (82%): m.p. 334.24 °C. ^1^H-NMR (CDCl_3_, 300 MHz) δ 9.05 (s, 2H, H4 and H4'), 8.22 (d, 2H, *J* = 8.4 Hz, H8 of q), 8.08 (d, 2H, *J* = 7.6 Hz, H5 of q), 7.91 (s, 2H, H4 of q), 7.75 (d, 2H, *J* = 8.1 Hz, H5 and H5'), 7.71 (td, 2H, *J* = 7.8, 1.4 Hz, H7 of q), 7.48 (td, 2H, *J* = 7.8, 1.4 Hz, H6 of q), 7.42 (td, 2H, *J* = 8.1, 1.5 Hz, H6 and H6'), 7.14 (td, 2H, *J* = 8.1, 1.2 Hz, H7 and H7'), 6.79 (d, 2H, *J* = 7.8 Hz, H8 and H8'), 6.67 (s, 2H, H1 and H1'), 3.05 (t, 4H, *J* = 5.8 Hz), 2.81 (t, 4H, *J* = 5.8 Hz). ^13^C-NMR (CDCl_3_, 62.5 MHz) δ 153.62, 150.73, 148.73, 147.70, 141.81, 141.19, 139.75, 134.63, 133.71, 130.72, 129.35, 128.73, 127.89, 127.75, 126.96, 126.09, 124.07, 123.59, 120.60, 117.65, 65.85, 28.76, 28.69. MS (ESI): *m*/*z* = 623 [M+H]^+^, Anal. Calcd for C_47_H_30_N_2_: C, 90.65; H, 4.86; N, 4.50. Found: C, 89.67; H, 5.03; N, 4.65.

*6,6',7,7'-Tetrahydro-9,9'-spirobi(indeno*[2',1':6,7]*naphtho*[1,2-b]*-1,8-naphthyridine)* (**7b**). The same procedure described above for **7a** but with 2-aminonicotinaldehyde (**4b**) afforded the desired product as pale yellow crystalline solid (78%): m.p. 303.658 °C. ^1^H-NMR (CDCl_3_, 250 MHz) δ 9.28 (s, 2H, H4 and H4'), 9.09 (dd, 2H, *J* = 4.3, 2.0 Hz, H7 of np), 8.11 (dd, 2H, *J* = 7.5, 2.0 Hz, H5 of np), 8.07 (d, 2H, *J* = 7.5 Hz, H5 and H5'), 7.92 (s, 2H, H4 of np), 7.438 (td, 2H, *J* = 7.5, 1.0 Hz, H6 of np), 7.437 (td, 2H, *J* = 8.1, 1.0 Hz, H6 and H6'), 7.14 (td, 2H, *J* = 7.5, 1.0 Hz, H7 and H7’), 6.80 (d, 2H, *J* = 7.5 Hz, H8 and H8'), 6.69 (s, 2H, H1 and H1'), 3.10 (t, 4H, *J* = 5.8 Hz), 2.87 (t, 4H, *J* = 5.8 Hz). ^13^C-NMR (CDCl_3_, 62.5 MHz) δ 156.77, 155.86, 152.84, 151.61, 148.62, 141.78, 141.38, 139.79, 136.14, 134.50, 134.08, 132.06, 128.15, 127.89, 124.14, 123.59, 122.34, 121.55, 120.75, 118.86, 60.37, 28.56, 28.46. MS (ESI): *m*/*z* = 625 [M+H]^+^, Anal. Calcd for C_45_H_28_N_4_: C, 86.51; H, 4.52; N, 8.97. Found: C, 86.67; H, 4.42; N, 9.05.

*8,8',9,9'-Tetrahydro-11,11'-spirobi(benzo[c]fluoreno*[3,2-h]*acridine)* (**7c**). The same procedure described above for **7a** but with 1-aminonaphthalene-2-carbaldehyde (**4c**) afforded the desired product as pale yellow crystalline solid (75%): m.p. 395.05 °C. ^1^H-NMR (CDCl_3_, 600 MHz) δ 9.63 (dd, 2H, *J* = 7.8, 1.0 Hz, H10 of bq), 9.25 (s, 2H, H4 and H4'), 8.18 (dd, 2H, *J* = 7.5, 1.0 Hz, H7 of bq), 7.95 (s, 2H, H4 of bq), 7.93 (d, 2H, *J* = 7.8 Hz, H5 and H5'), 7.84 (td, 2H, *J* = 7.8, 1.0 Hz, H9 of bq), 7.79 (d, 2H, *J* = 8.4 Hz, H5/H6 of bq), 7.73 (td, 2H, *J* = 7.8, 1.0 Hz, H8 of bq), 7.67 (d, 2H, *J* = 8.4 Hz, H6/H5 of bq), 7.49 (td, 2H, *J* = 7.7, 1.0 Hz, H6 and H6'), 7.19 (td, 2H, *J* = 7.7, 1.0 Hz, H7 and H7'), 6.87 (d, 2H, *J* = 7.7 Hz, H8 and H8'), 6.74 (s, 2H, H1 and H1'), 3.10 (t, 4H, *J* = 5.8 Hz), 2.87 (t, 4H, *J* = 5.8 Hz). ^13^C-NMR (CDCl_3_, 62.5 MHz) δ 151.84, 150.52, 148.84, 145.26, 141.94, 141.11, 139.42, 135.06, 134.13, 133.53, 131.84, 130.97, 127.91, 127.83, 127.77 (two C’s), 127.25, 126.85, 125.75, 125.03, 124.52, 124.18, 123.64, 120.41, 117.45, 65.87, 28.66, 28.44. MS (ESI): *m*/*z* = 723 [M+H]^+^, Anal. Calcd for C_55_H_34_N_4_**∙**H_2_O: C, 89.16; H, 4.90; N, 3.78. Found: C, 89.67; H, 4.92; N, 3.91.

*8,8',9,9'-Tetrahydro-11,11'-spirobi(indeno[2',1':6,7]naphtho[1,2-b]-1,10-phenanthroline)* (**7d**). The same procedure described above for **7a** but with 8-aminoquinoline-7-carbaldehyde (**4d**) afforded the desired product as pale yellow crystalline solid (80%): m.p. 266.77 °C. ^1^H-NMR (CDCl_3_, 600 MHz) δ 9.38 (s, 2H, H4 and H4'), 9.28 (dd, 2H, *J* = 4.5, 1.8 Hz, H9 of phen), 8.26 (dd, 2H, *J* = 8.1, 1.8 Hz, H7 of phen), 8.21 (d, 2H, *J* = 7.5 Hz, H5 and H5'), 8.01 (s, 2H, H4 of phen), 7.78–7.72 (AB quartet, 4H, H5 and H6 of phen), 7.65 (dd, 2H, *J* = 8.1, 4.5 Hz, H8 of phen), 7.43 (td, 2H, *J* = 7.5, 1.5 Hz, H6 and H6'), 7.14 (td, 2H, *J* = 7.5, 1.5 Hz, H7 and H7'), 6.80 (d, 2H, *J* = 7.5 Hz, H8 and H8'), 6.68 (s, 2H, H1 and H1'), 3.12 (t, 4H, *J* = 6.3 Hz), 2.85 (t, 4H, *J* = 6.3 Hz). ^13^C-NMR (CDCl_3_, 62.5 MHz) δ 151156.76, 155.83, 152.80, 151.47, 148.89, 148.52, 141.74, 141.46, 139.64, 136.11, 134.46, 133.98, 131.01, 128.04, 127.89, 127.77, 124.27, 123.86,, 123.33, 122.31, 121.50, 120.70, 119.99, 118.83, 65.97, 28.54, 28.44. MS (ESI): *m*/*z* = 725 [M+H]^+^, Anal. Calcd for C_53_H_32_N_4_**∙**0.75H_2_O: C, 86.21; H, 4.57; N, 7.59. Found: C, 86.87; H, 4.62; N, 7.65.

## 4. Conclusions

A series of 9,9'-spirobifluorene-derived *N*-heterocycles were prepared from 8,9-dihydrospiro-(benzo[*b*]fluorene-11,9'-fluoren)-6(7*H*)-one and 8,8',9,9'-tetrahydro-11,11'-spirobi(benzo[*b*]-fluorene)-6,6'(7*H*,7'*H*)-dione and 2-aminoarenecarbaldehydes such as 2-aminobenzaldehyde, 2-amino- nicotinealdehyde, 1-amino-2-naphthaldehyde, and 8-aminoquinoline-7-carbaldehyde, respectively. The monoheteroaryl series was crystalline in nature, while diheroaryl ones showed an amorphous nature. In addition to the absorptions based on the parent 9,9'-spirobifluorene skeleton in the 225–234, 239–280, 296–298, and 308–328 nm regions, absorptions due to the π-π* transition of heterocycles were observed in the 351–375 nm region. All compounds showed strong photoluminescence in the 390–430 nm region regardless the excitation wavelength. 

## Supplementary Materials

Supplementary materials can be accessed at: http://www.mdpi.com/1420-3049/18/11/13680/s1.

## References

[1] Clarkson R.G., Gomberg M. (1930). Spirans with four aromaatic radicals on the spiro carbon atom. J. Am. Chem. Soc..

[2] Haas G., Prelog V. (1969). Optisch aktive 9,9'-Spirobifluoren-Derivate. Helv. Chim. Acta.

[3] Prelog V., Kovačević M., Egli M. (1989). Liphophilic tartaric acid esters as enantioselective ionophores. Angew. Chem. Int. Ed. Engl..

[4] Alcázar Montero V., Tomlinson L., Houk K.N., Diederich F. (1991). Selective α,ω-dicarboxylic acid recognition in a chiral cleft shaped by the 9,9'-spirobifluorene unit. Tetrahedron Lett..

[5] Alcázar V., Diederich F. (1992). Enantioselective complexation of chiral dicarboxylic acids in clefts of functionalized 9,9'-spirobifluorenes. Angew. Chem. Int. Ed. Engl..

[6] Diederich F., Felber B. (2002). Supramolecular chemistry of dendrimers with functional cores. Proc. Natl. Acad. Sci. USA.

[7] Weber E., Ahrendt J., Czugler M., Csöregh I. (1986). Selective inclusion and separation of isomeric homologous hydrocarbons by hydrocarbon host lattices. Angew. Chem. Int. Ed. Engl..

[8] Das G., Hamilton A.D. (1997). Carbohydrate recognition: Enantioselective spirobifluorene diphosphonate receptors. Tetrahedron Lett..

[9] Hernández J.V., Almaraz M., Raposo C., Martín M., Lithgow A., Crego M., Caballero C., Morán J.R. (1998). Chiral recognition of tartaric acid derivatives with chromenone-benzoisoxazole receptors with a spirobifluorene spacer. Tetrahedron Lett..

[10] Tejeda A., Oliva A.I., Simón L., Grande M., Caballero C., Morán J.R. (2000). A macrocyclic receptor for the chiral recognition of hydroxycarboxylates. Tetrahedron Lett..

[11] Poriel C., Rault-Berthelot J., Thirion D. (2013). Modulation of the electronic properties of 3*π*-2spiro compounds derived from bridged oligophenylenes: A structure-property relationship. J. Org. Chem..

[12] Huang J., Yang X., Wang J., Zhong C., Wang L., Qin J., Li Z. (2012). New tetraphenylethene-based efficient blue luminophors: Aggregation induced emission and partially controllable emitting color. J. Mater. Chem..

[13] Müller C.D., Falcou A., Reckefuss N., Rojahn M., Wiederhirn V., Rudati P., Frohne H., Nuyken O., Becker H., Meerholz K. (2003). Multicolor organic light-emitting displays by solution processing. Nature.

[14] Grimsdale A.C., Chan K.L., Martin R.E., Jokisz P.G., Holmes A.B. (2009). Synthesis of light-emitting conjugated polymers for applications in electroluminescent devices. Chem. Rev..

[15] Omer K.M., Ku S.-Y., Wong K.-T., Bard A.J. (2009). Green electrogenerated chemluminescence of highly fluorescent benzothiadiazole and fluorene derivatives. J. Am. Chem. Soc..

[16] Saragi T.P.I., Spehr T., Siebert A., Fuhrmann-Lieker T., Salbeck J. (2007). Spiro compounds for organic optoelectronics. Chem. Rev..

[17] Moreau F., Audebrand N., Poriel C., Moizan-Baslé V., Ouvry J. (2011). A 9,9′-spirobifluorene based metal–organic framework: Synthesis, structure analysis and gas sorption properties. J. Mater. Chem..

[18] Ferrand Y., Poriel C., Le Maux P., Rault-Berthelot J., Simonneaux G. (2005). Asymmetric heterogeneous carbene transfer catalyzed by optically active ruthenium spirobifluorenylporphyrin polymers. Tetrahedron Asymmetry.

[19] Murase S., Tominaga T., Kitazawa D. (2005). Organic Electroluminescent Device. Japan Patent.

[20] Wu F., Riesgo E.C., Thummel R.P., Juris A., Hissler M., Elghayoury A., Ziessel R. (1999). Closely-spaced chelating centers: Synthesis of novel spiro-bridged bis-phenathrolines and bis-indole derivatives. Tetrahedron Lett..

[21] Liao L.-S., Begley W.J., Pellow C.A. (2009). Phosphorscent OLED having double-blocking layers having different triplet energies. U.S. Patens Appl. Publ..

[22] Tominaga T., Kitazawa D., Makiyama A., Kohama A. (2005). Light-emitting device material and light-emitting devices. PCT Int. Appl. WO.

[23] Jahng Y., Rahman A.F.M.M. (2010). Synthesis and properties of 2,2'-Di(heteroaryl)-9,9'-spirobifluorenes. Bull. Chem. Soc. Jpn..

[24] Qu Y.-W., Shi K.-H., Liu Q.-C. (2008). Synthesis of quinolinyl spirobifluorene derivatives. Chin. J. Synth. Chem..

[25] Chea J.M., Jahng Y. (2009). Synthesis and properties of 9,9'-spirobifluorene-based heterocycles. Heterocycles.

[26] Opie J.W., Smith L.I. (1955). *o*-Aminobenzaldehyde. Org. Synth..

[27] Majewicz G., Caluwe O.A. (1974). Facile synthesis of 2-aminonicotinaldehyde. J. Org. Chem..

[28] Riesgo E.C., Jin X., Thummel R.P. (1996). Introduction of benzo[*h*]quinoline and 1,10-phenanthroline subunits by friedländer methodology. J. Org. Chem..

[29] Thirion D., Poriel C., Métivier R., Rault-Berthelot J., Barrière F., Jeannin O. (2011). Violet-to-blue tunable emission of aryl-substituted dispirofluorene–indenofluorene isomers by conformationally-controllable intramolecular excimer formation. Chem. Eur. J..

[30] Pretsch E., Seibl J., Simon W. (1989). Tables of Spectral Data for Structural Determination of Organic Compounds.

