# Supplementary material for: Synthesis and Properties of Annulated 2-(Azaar-2-yl)- and 2,2'-Di(azaar-2-yl)-9,9'-spirobifluorenes
Source: Molecules. 2013 Nov 5;18(11):13680–90. doi: 10.3390/molecules181113680 (PMC6270136; doi:10.3390/molecules181113680)
Supplement: Supplementary file 1 [file molecules-18-13680-s001.pdf]

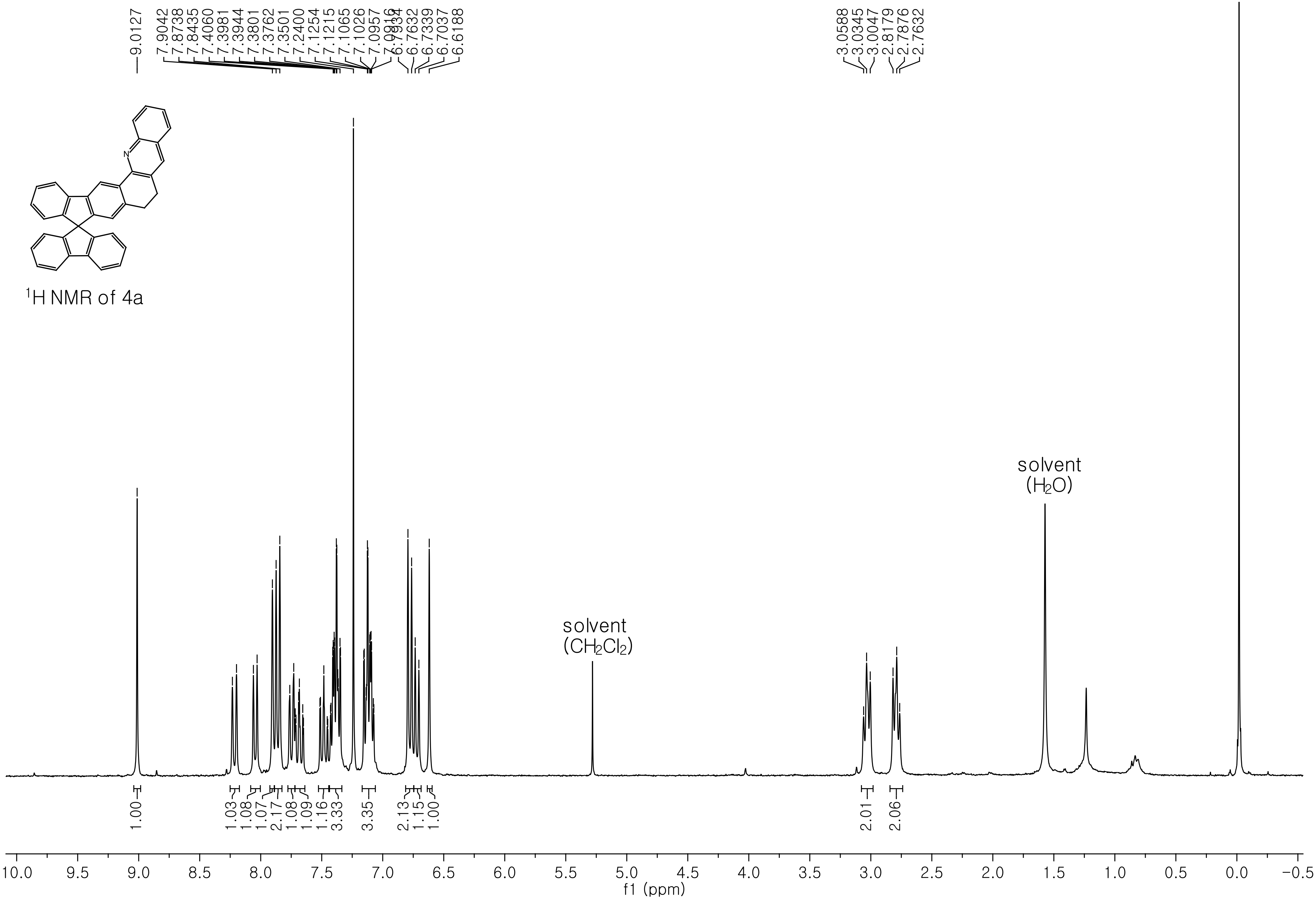
9.0127
7.9042
7.8738
7.8435
7.4060
7.3981
7.3944
7.3801
7.3762
7.3501
7.2400
7.1254
7.1215
7.1065
7.1026
7.0957
6.7934
6.7632
6.7339
6.7037
6.6188
3.0588
3.0345
3.0047
2.8179
2.7876
2.7632
N
solvent
(CH_2Cl_2)
solvent
(H_2O)
1.00
1.03
1.08
1.07
2.17
1.08
1.09
1.16
3.33
3.35
2.13
1.15
1.00
2.01
2.06
10.0
9.5
9.0
8.5
8.0
7.5
7.0
6.5
6.0
5.5
5.0
4.5
4.0
3.5
3.0
2.5
2.0
1.5
1.0
0.5
0.0
−0.5
f1 (ppm)

^{1}H NMR of 4a

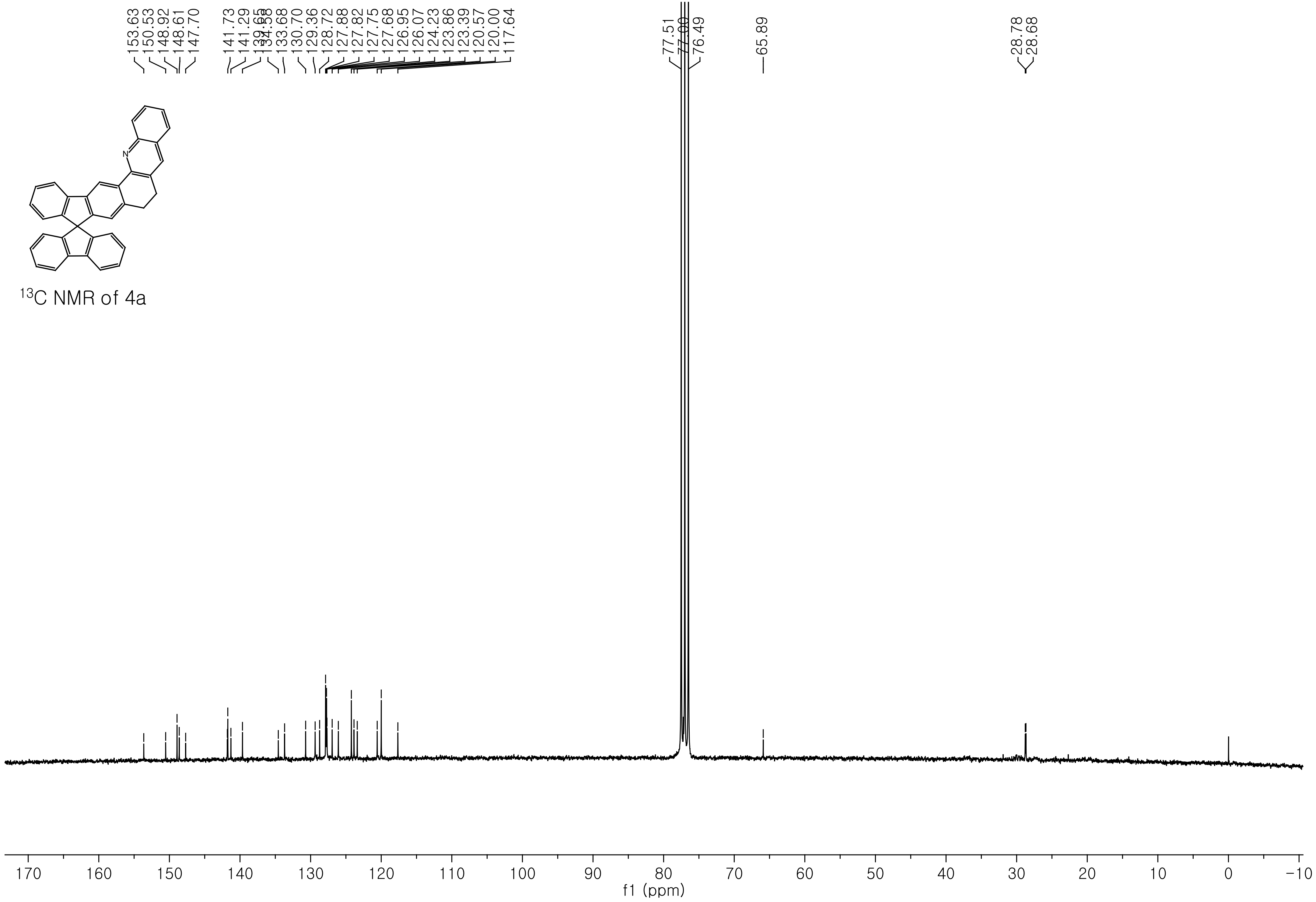
153.63
150.53
148.92
148.61
147.70
141.73
141.29
139.65
134.58
133.68
130.70
129.36
128.72
127.88
127.82
127.75
127.68
126.95
126.07
124.23
123.86
123.39
120.57
120.00
117.64
77.51
77.00
76.49
65.89
28.78
28.68
N
13C NMR of 4a
170 160 150 140 130 120 110 100 90 80 70 60 50 40 30 20 10 0 −10
f1 (ppm)

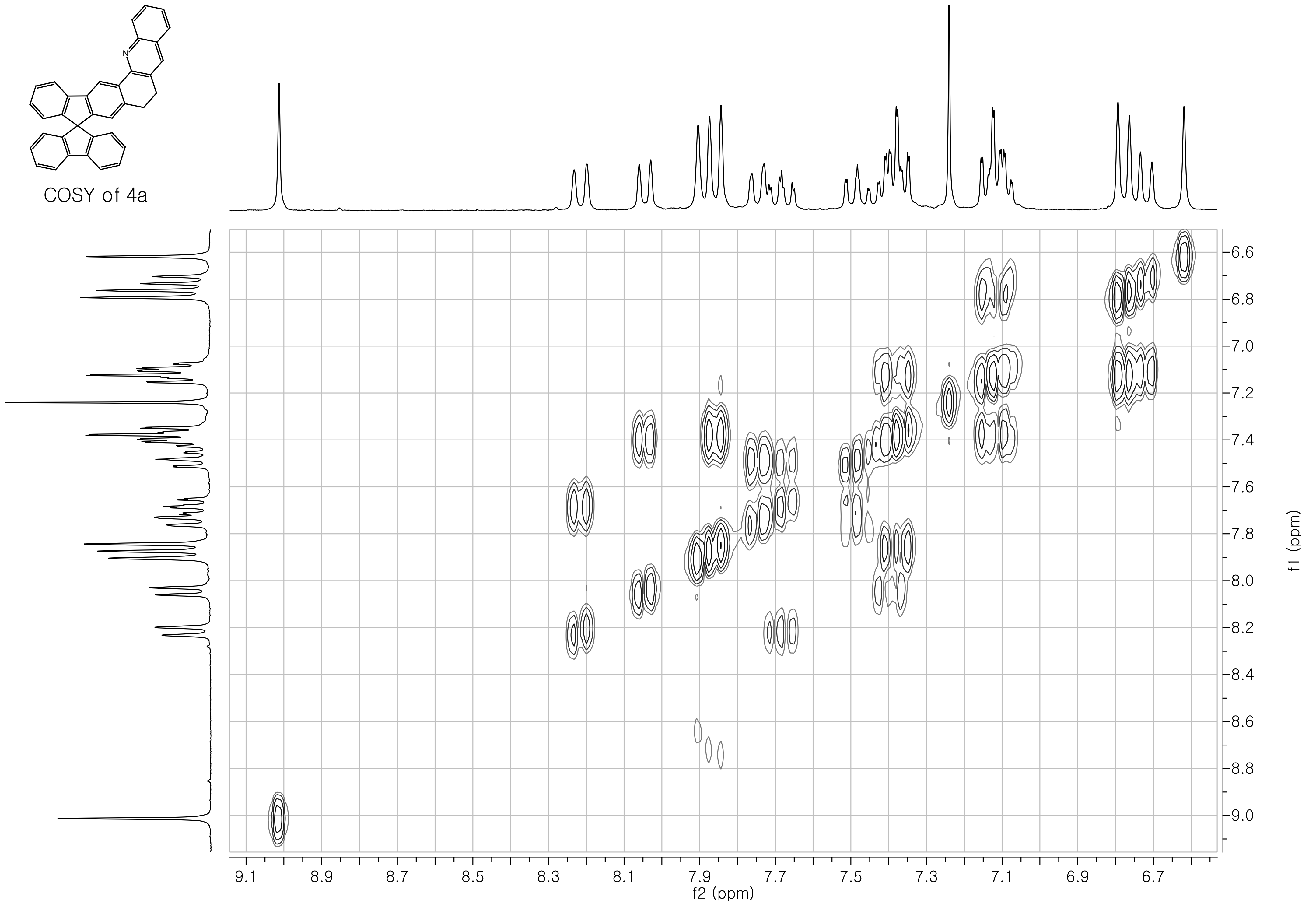
COSY of 4a
f2 (ppm)
f1 (ppm)

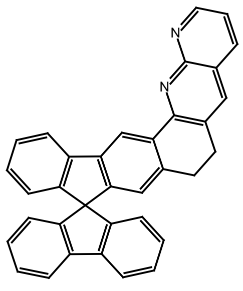

^{1}H NMR of 4b

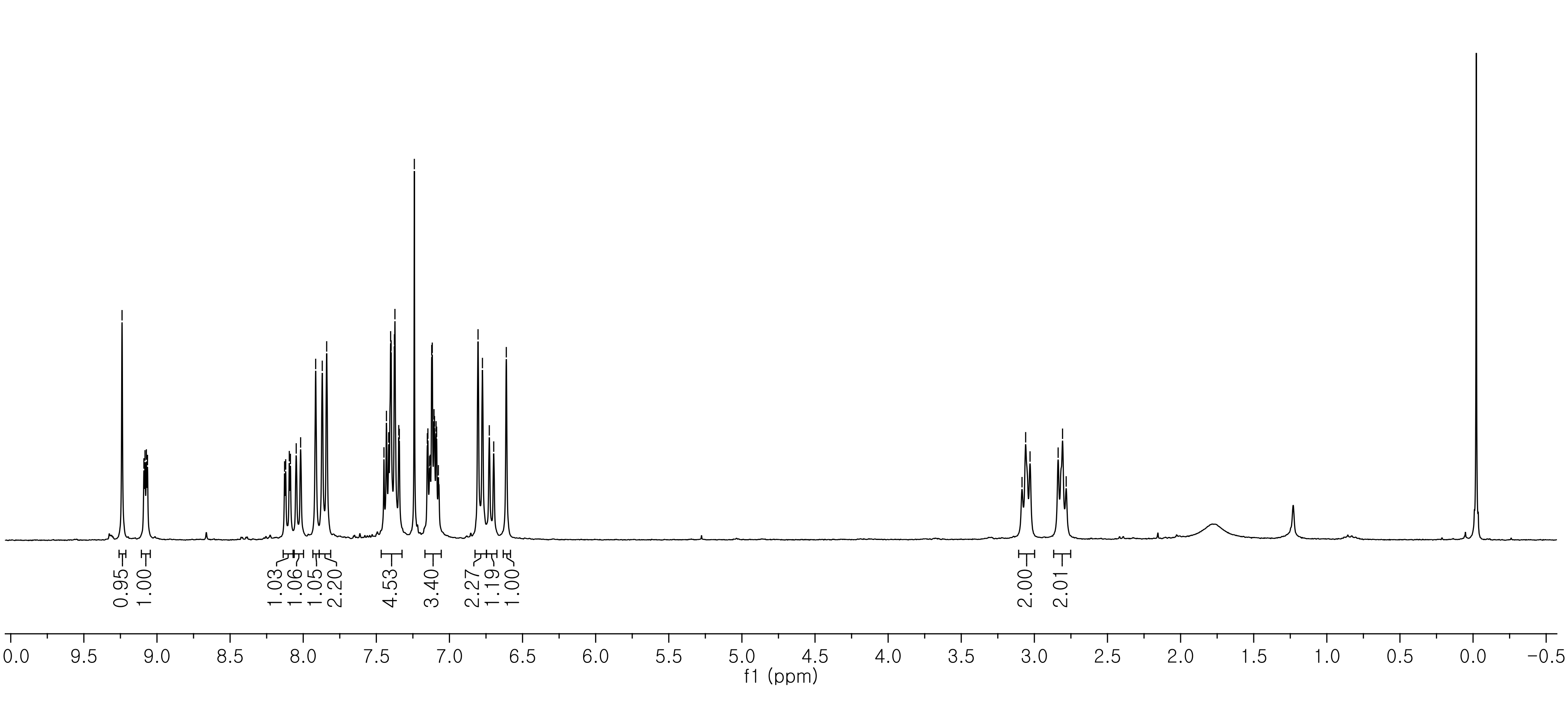
9.2389
9.0894
9.0818
9.0725
9.0648
8.1197
8.0951
8.0874
8.0478
8.0175
7.9148
7.8697
7.8395
7.4478
7.4307
7.4155
7.4022
7.3988
7.3764
7.3727
7.3466
7.3428
7.2400
7.1513
7.1474
7.1355
7.1316
7.1212
7.1174
7.1055
7.1020
7.0915
7.0876
7.0758
6.8044
6.7742
6.7274
6.6972
6.6112
3.0845
3.0595
3.0296
2.8375
2.8073
2.7824
0.95
1.00
1.03
1.06
1.05
2.20
4.53
3.40
2.27
1.19
1.00
2.00
2.01
10.0
9.5
9.0
8.5
8.0
7.5
7.0
6.5
6.0
5.5
5.0
4.5
4.0
3.5
3.0
2.5
2.0
1.5
1.0
0.5
0.0
−0.5
f1 (ppm)

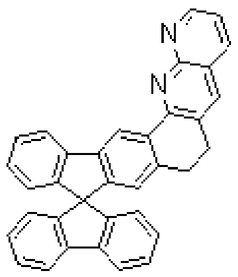

^{13}C NMR of 4b

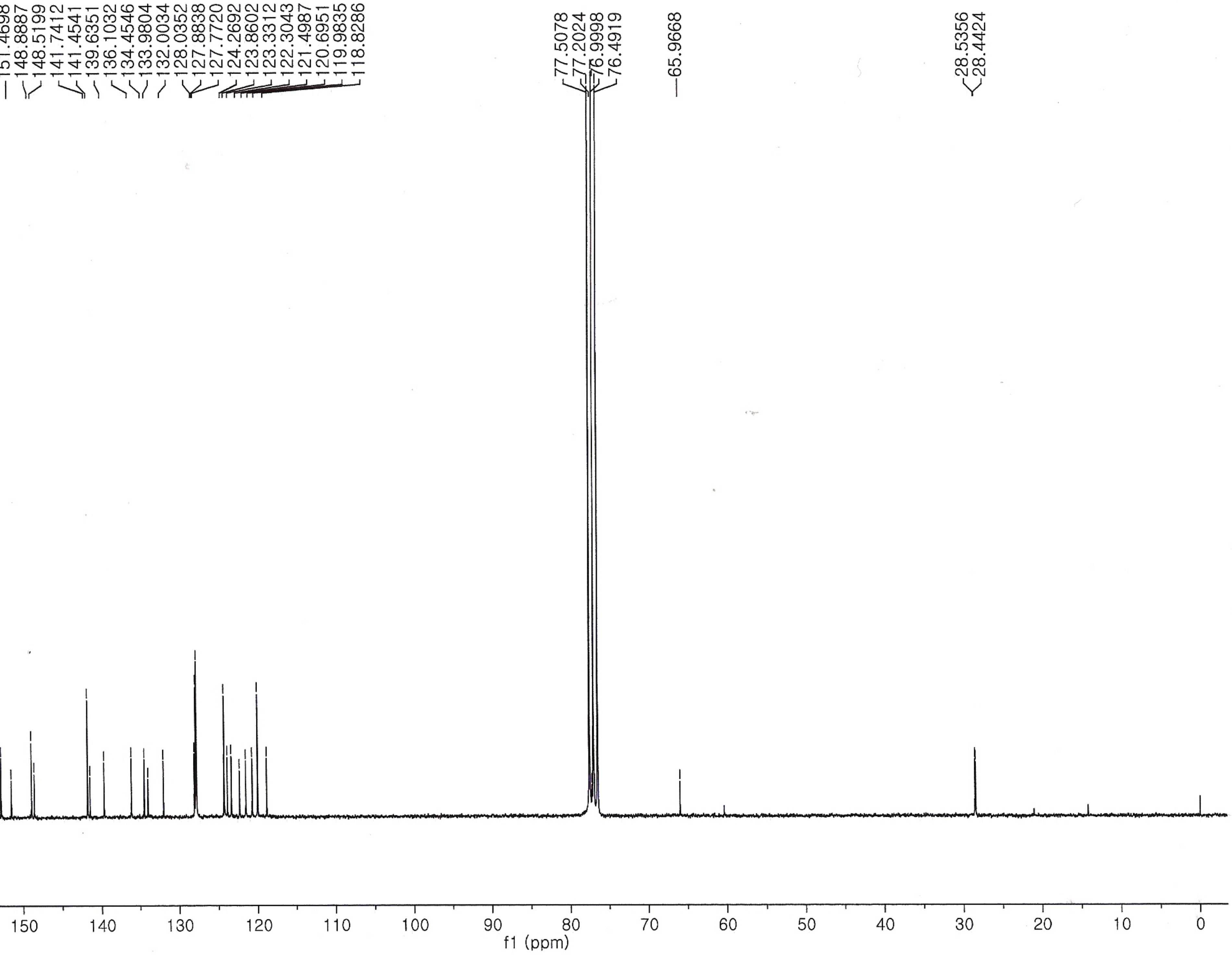
156.7549
155.8238
152.7964
151.4698
148.8887
148.5199
141.7412
141.4541
139.6351
136.1032
134.4546
133.9804
132.0034
128.0352
127.8838
127.7720
124.2692
123.8602
123.3312
122.3043
121.4987
120.6951
119.9835
118.8286
77.5078
77.2024
76.9998
76.4919
65.9668
28.5356
28.4424
170 160 150 140 130 120 110 100 90 80 70 60 50 40 30 20 10 0
f1 (ppm)

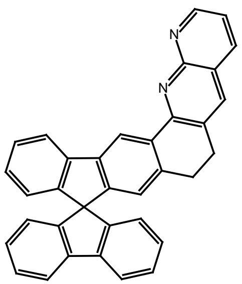
N
N

COSY of 4b

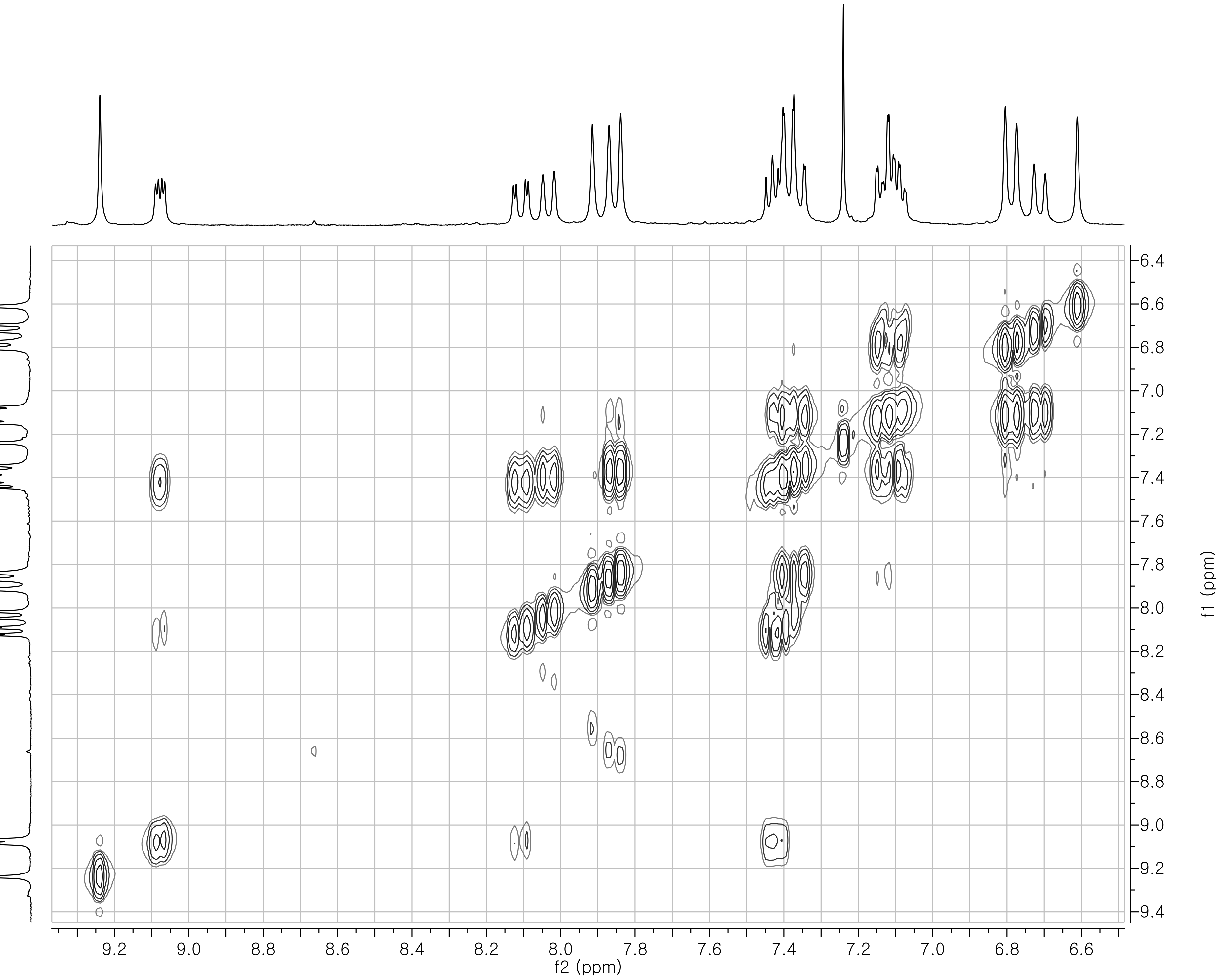
f2 (ppm)
f1 (ppm)
9.2 9.0 8.8 8.6 8.4 8.2 8.0 7.8 7.6 7.4 7.2 7.0 6.8 6.6
6.4 6.6 6.8 7.0 7.2 7.4 7.6 7.8 8.0 8.2 8.4 8.6 8.8 9.0 9.2 9.4

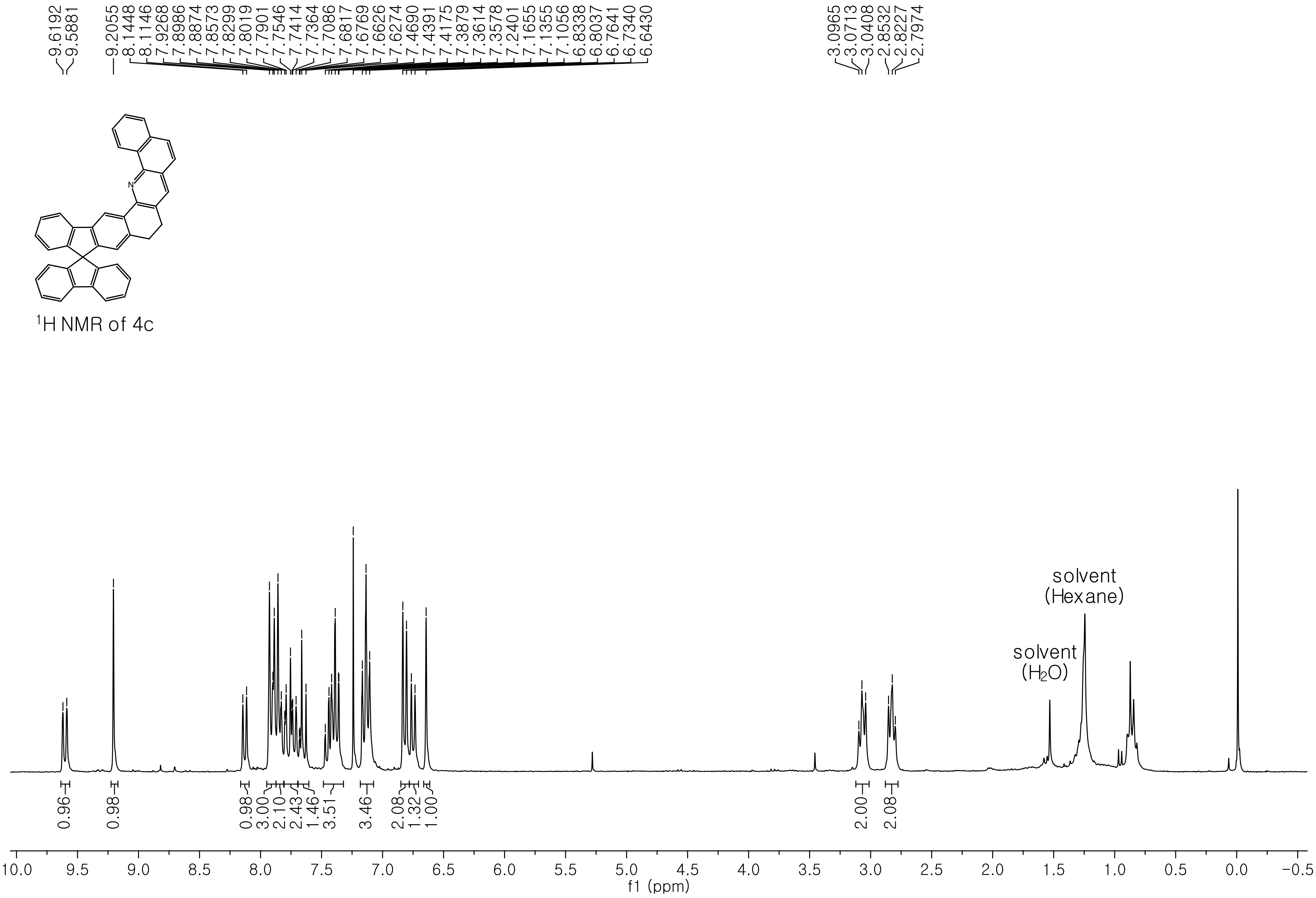

9.6192
9.5881
9.2055
8.1448
8.1146
7.9268
7.8986
7.8874
7.8573
7.8299
7.8019
7.7901
7.7546
7.7414
7.7364
7.7086
7.6817
7.6769
7.6626
7.6274
7.4690
7.4391
7.4175
7.3879
7.3614
7.3578
7.2401
7.1655
7.1355
7.1056
6.8338
6.8037
6.7641
6.7340
6.6430
3.0965
3.0713
3.0408
2.8532
2.8227
2.7974
N
solvent
(Hexane)
solvent
(H_2O)
0.96
0.98
0.98
3.00
2.10
2.43
1.46
3.51
3.46
2.08
1.32
1.00
2.00
2.08
10.0 9.5 9.0 8.5 8.0 7.5 7.0 6.5 6.0 5.5 5.0 4.5 4.0 3.5 3.0 2.5 2.0 1.5 1.0 0.5 0.0 −0.5
f1 (ppm)

^{1}H NMR of 4c

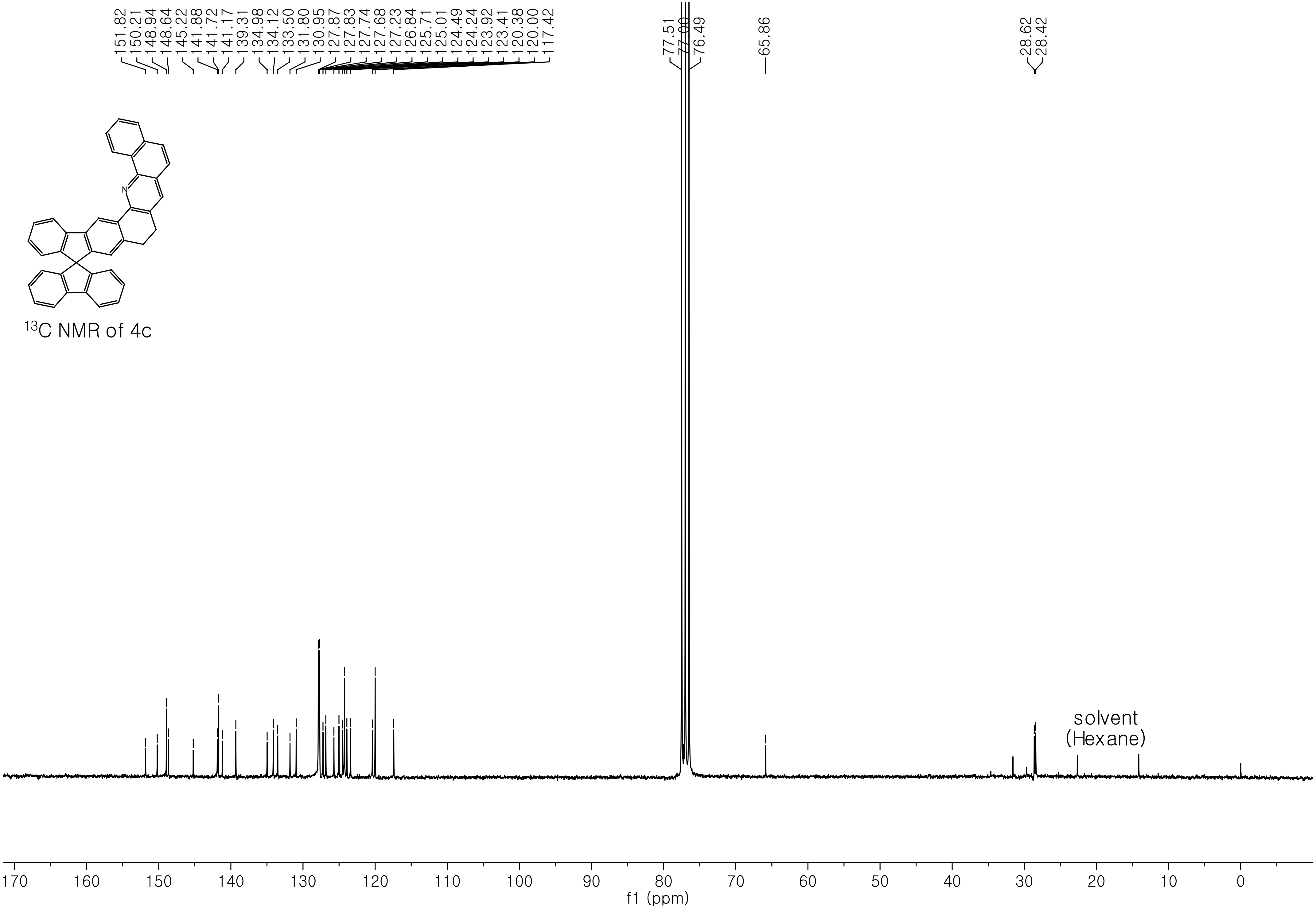

151.82
150.21
148.94
148.64
145.22
141.88
141.72
141.17
139.31
134.98
134.12
133.50
131.80
130.95
127.87
127.83
127.74
127.68
127.23
126.84
125.71
125.01
124.49
124.24
123.92
123.41
120.38
120.00
117.42
77.51
77.00
76.49
65.86
28.62
28.42
N
^{13}C NMR of 4c
solvent
(Hexane)
170 160 150 140 130 120 110 100 90 80 70 60 50 40 30 20 10 0
f1 (ppm)

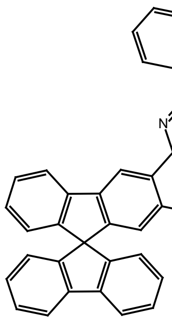
N

COSY of 4c

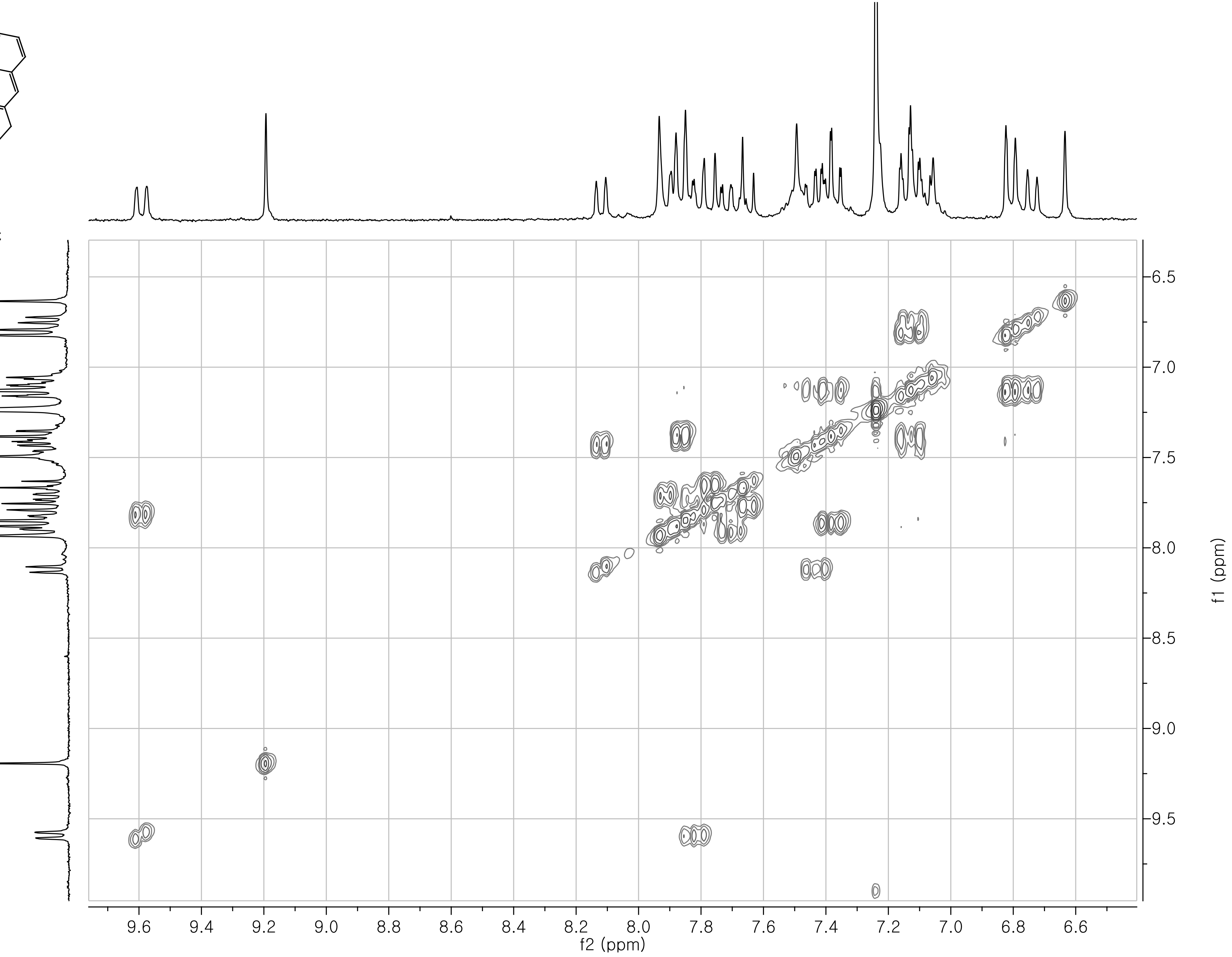
9.6
9.4
9.2
9.0
8.8
8.6
8.4
8.2
8.0
7.8
7.6
7.4
7.2
7.0
6.8
6.6
f2 (ppm)
6.5
7.0
7.5
8.0
8.5
9.0
9.5
f1 (ppm)

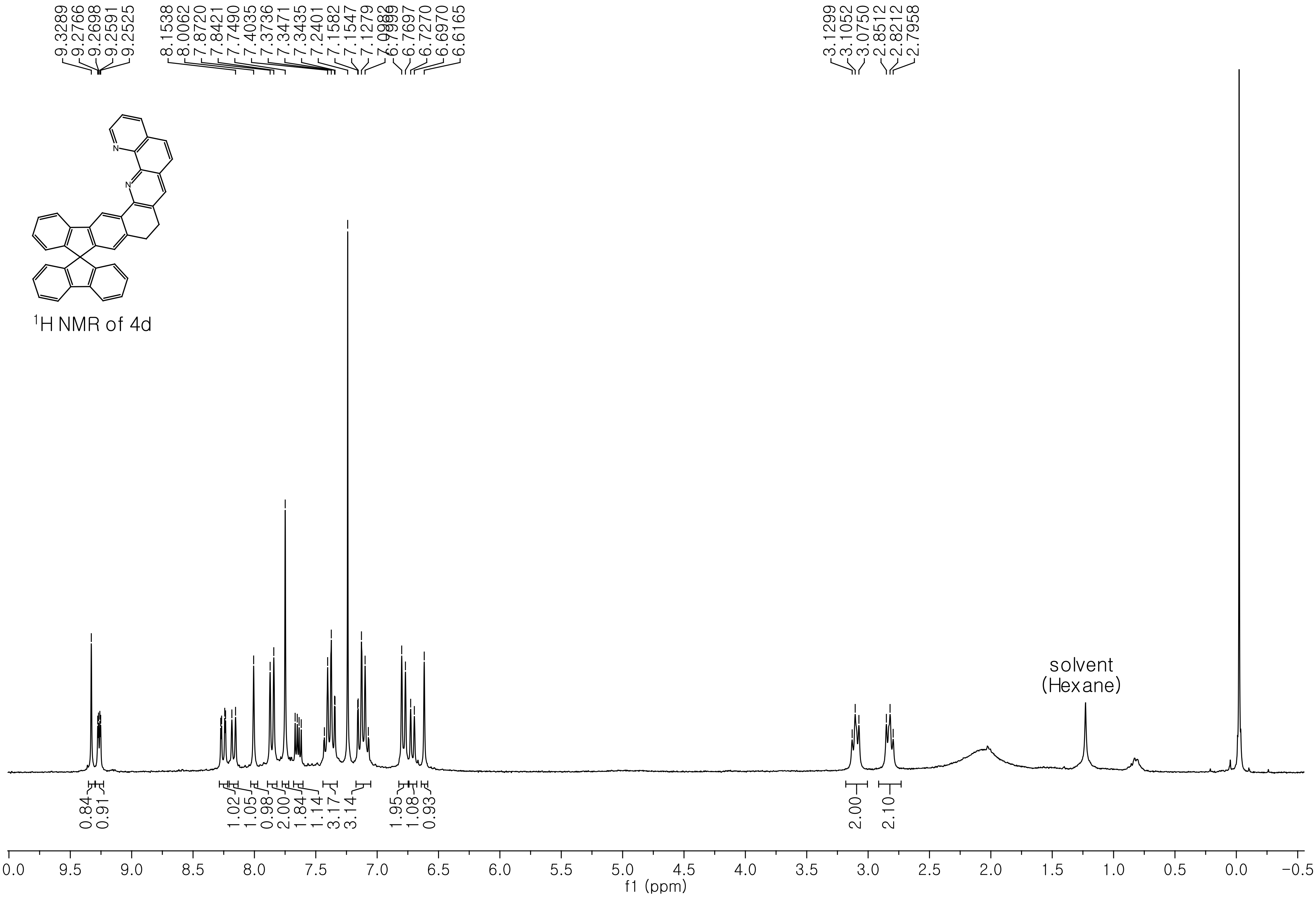
9.3289
9.2766
9.2698
9.2591
9.2525
8.1538
8.0062
7.8720
7.8421
7.7490
7.4035
7.3736
7.3471
7.3435
7.2401
7.1582
7.1547
7.1279
7.0982
6.7999
6.7697
6.7270
6.6970
6.6165
3.1299
3.1052
3.0750
2.8512
2.8212
2.7958
N
N
solvent
(Hexane)
0.84
0.91
1.02
1.05
0.98
2.00
1.84
1.14
3.17
3.14
1.95
1.08
0.93
2.00
2.10
10.0 9.5 9.0 8.5 8.0 7.5 7.0 6.5 6.0 5.5 5.0 4.5 4.0 3.5 3.0 2.5 2.0 1.5 1.0 0.5 0.0 −0.5
f1 (ppm)

^{1}H NMR of 4d

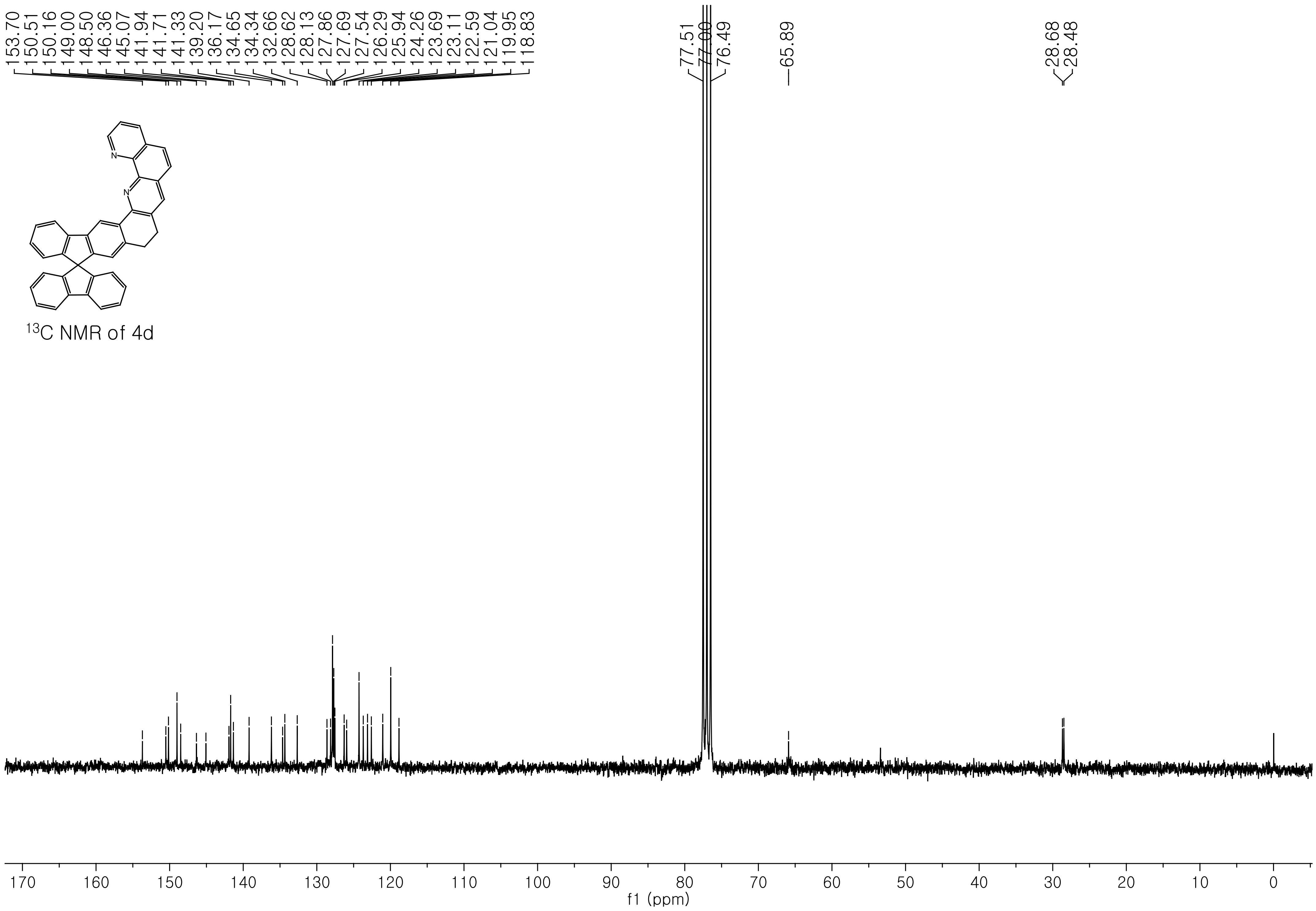
153.70
150.51
150.16
149.00
148.50
146.36
145.07
141.94
141.71
141.33
139.20
136.17
134.65
134.34
132.66
128.62
128.13
127.86
127.69
127.54
126.29
125.94
124.26
123.69
123.11
122.59
121.04
119.95
118.83
77.51
77.00
76.49
65.89
28.68
28.48
N
N
^{13}C NMR of 4d
170
160
150
140
130
120
110
100
90
80
70
60
50
40
30
20
10
0
f1 (ppm)

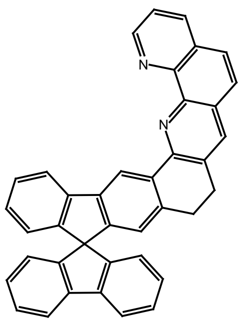
N
N

COSY of 4d

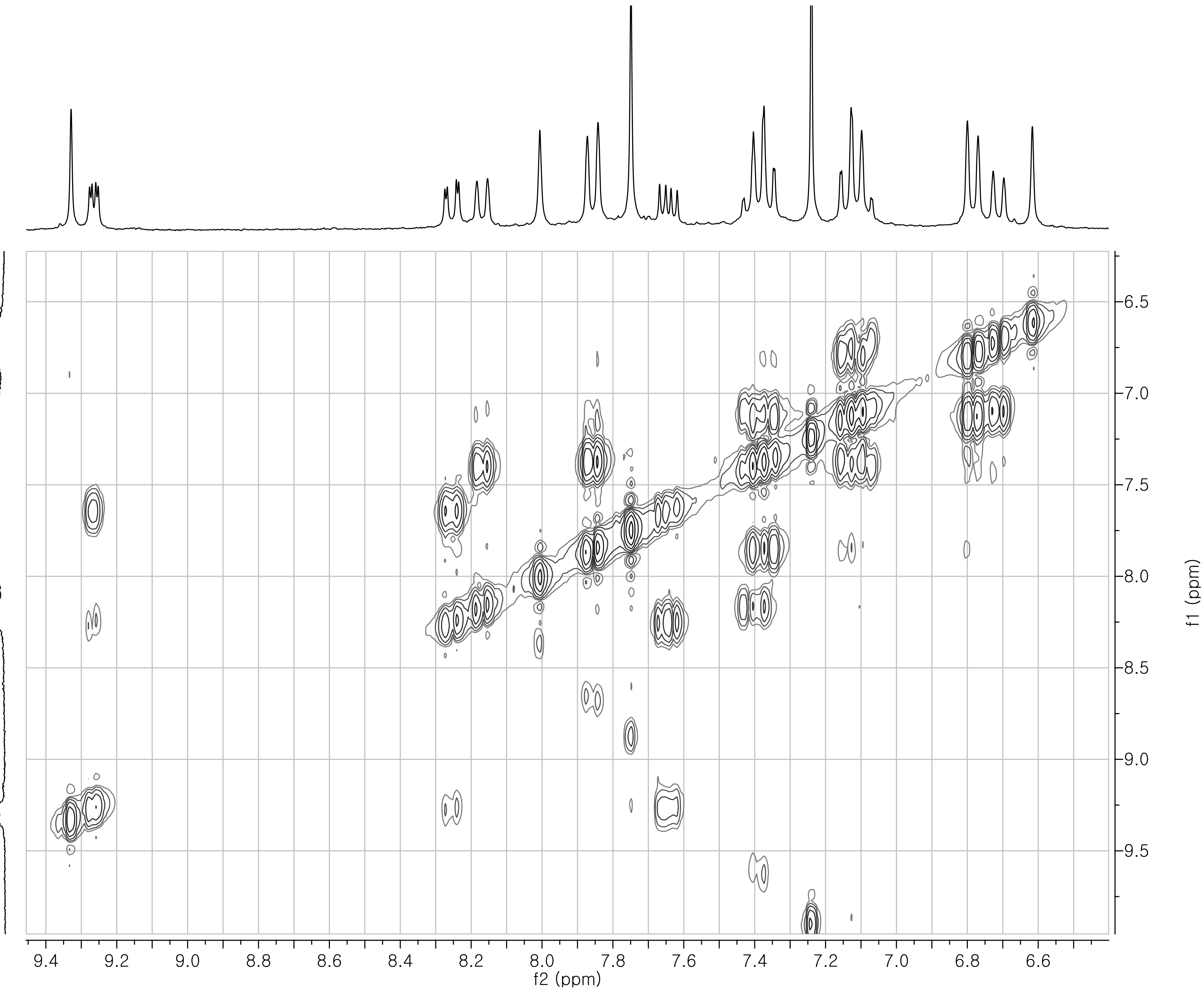
f2 (ppm)
f1 (ppm)
9.4
9.2
9.0
8.8
8.6
8.4
8.2
8.0
7.8
7.6
7.4
7.2
7.0
6.8
6.6
6.5
7.0
7.5
8.0
8.5
9.0
9.5

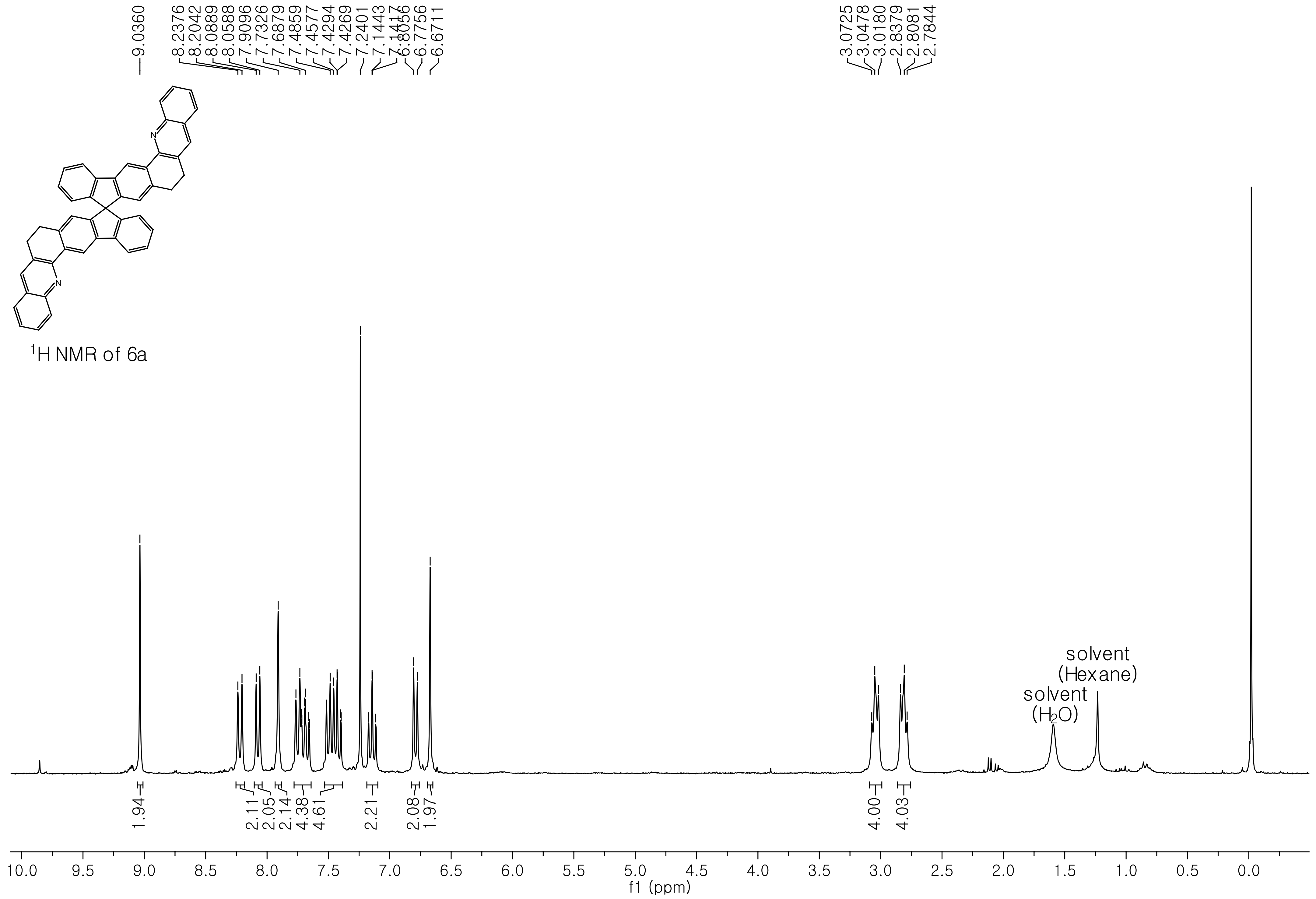

9.0360
8.2376
8.2042
8.0889
8.0588
7.9096
7.7326
7.6879
7.4859
7.4577
7.4294
7.4269
7.2401
7.1443
7.1417
6.8056
6.7756
6.6711
3.0725
3.0478
3.0180
2.8379
2.8081
2.7844
N
N
¹H NMR of 6a
solvent
(Hexane)
solvent
(H_2O)
1.94
2.11
2.05
2.14
4.38
4.61
2.21
2.08
1.97
4.00
4.03
10.0
9.5
9.0
8.5
8.0
7.5
7.0
6.5
6.0
5.5
5.0
4.5
4.0
3.5
3.0
2.5
2.0
1.5
1.0
0.5
0.0
f1 (ppm)

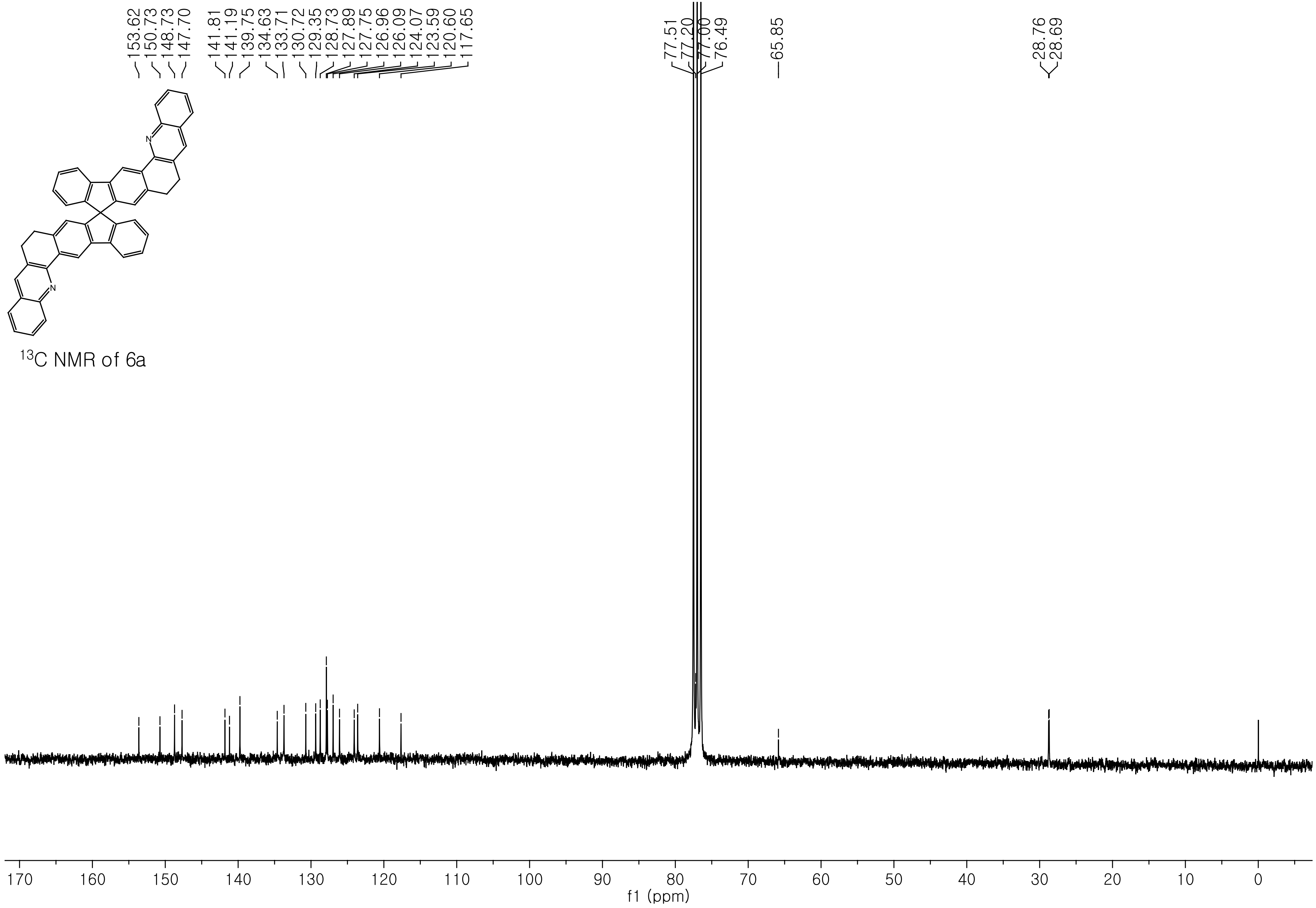
153.62
150.73
148.73
147.70
141.81
141.19
139.75
134.63
133.71
130.72
129.35
128.73
127.89
127.75
126.96
126.09
124.07
123.59
120.60
117.65
77.51
77.20
77.00
76.49
65.85
28.76
28.69
N
N
^{13}C NMR of 6a
170
160
150
140
130
120
110
100
90
80
70
60
50
40
30
20
10
0
f1 (ppm)

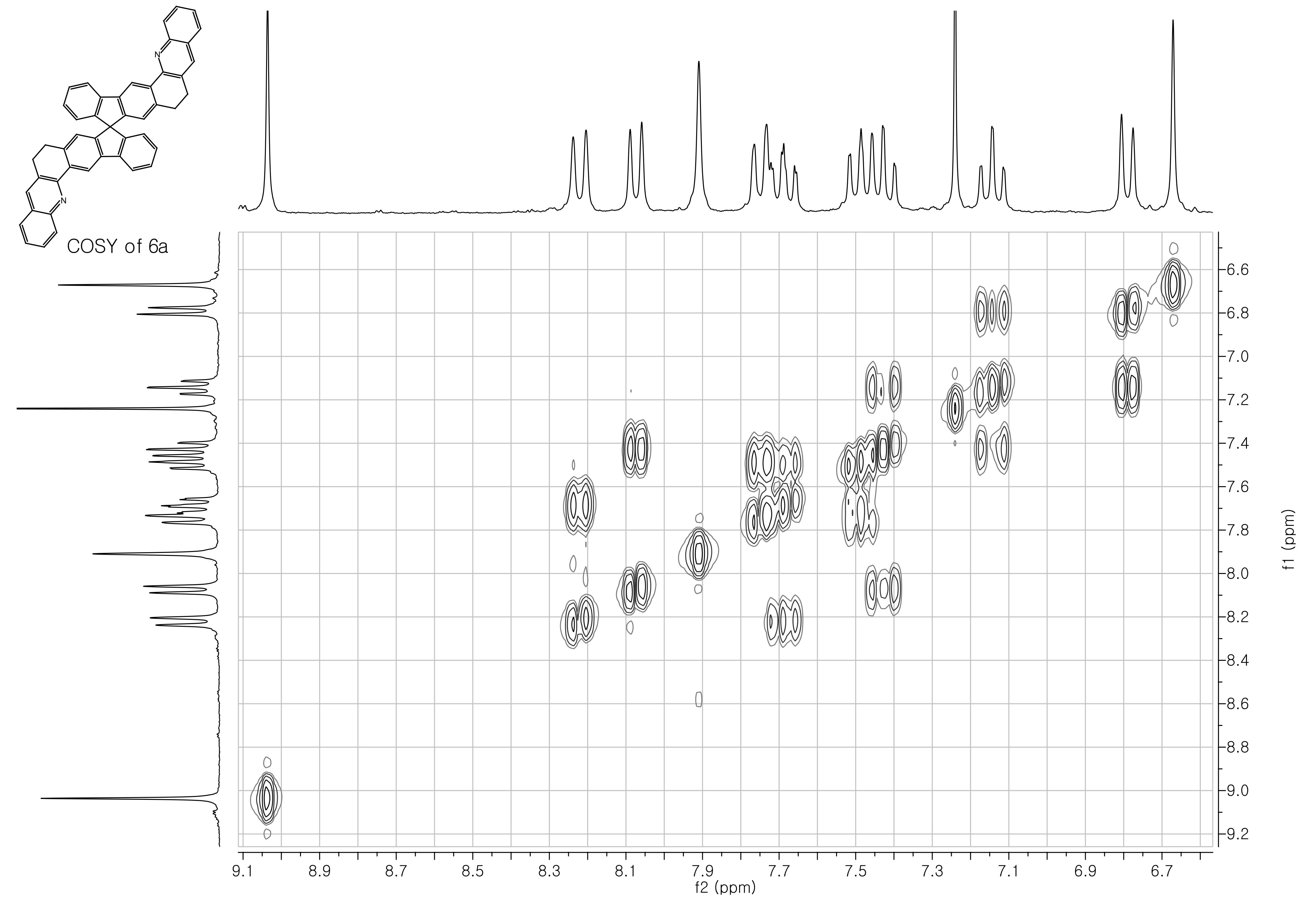

N
N
COSY of 6a
f2 (ppm)
f1 (ppm)
9.1
8.9
8.7
8.5
8.3
8.1
7.9
7.7
7.5
7.3
7.1
6.9
6.7
6.6
6.8
7.0
7.2
7.4
7.6
7.8
8.0
8.2
8.4
8.6
8.8
9.0
9.2

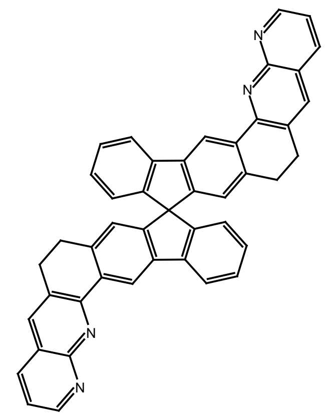
N
N
N
N

^{1}H NMR of 6b

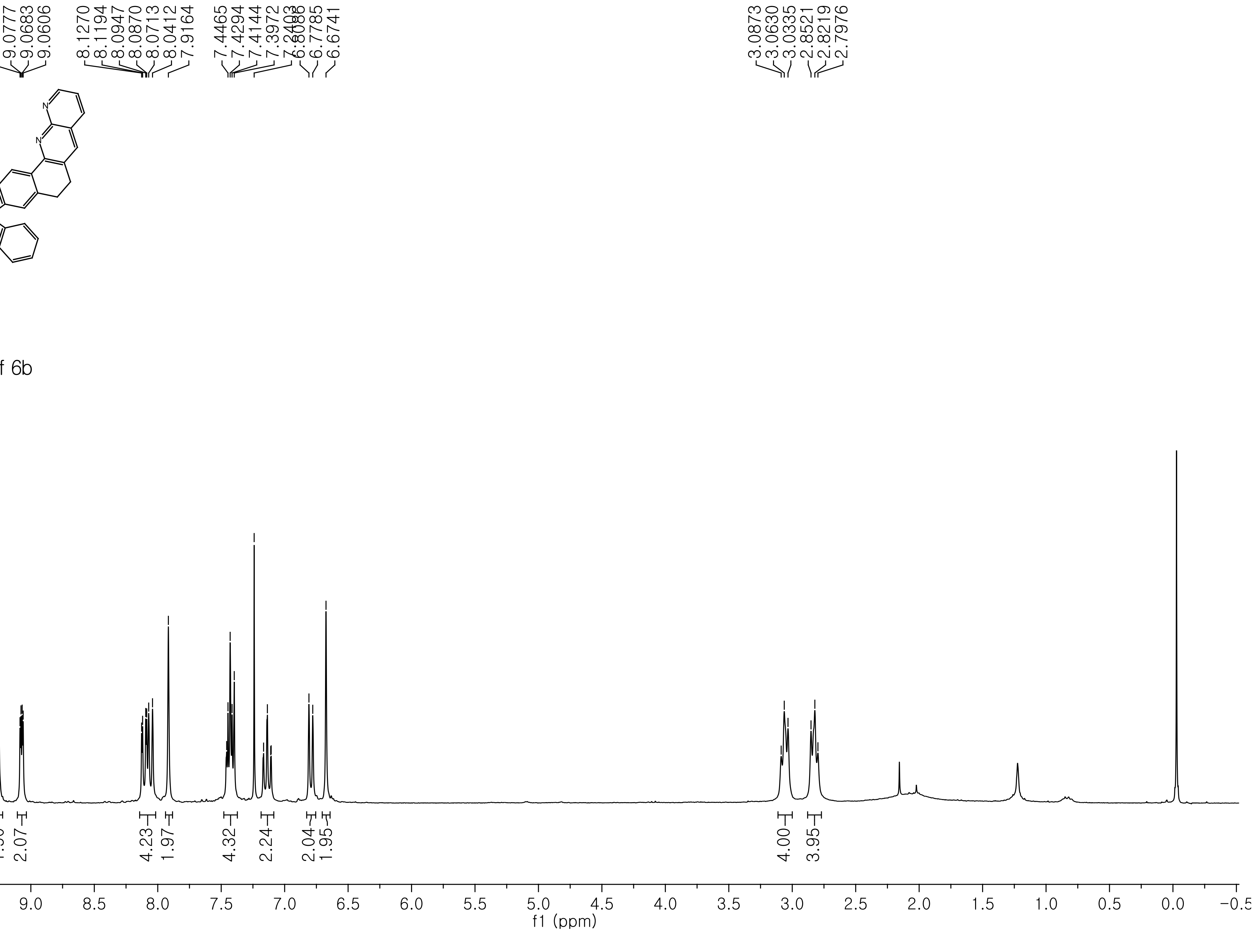
9.2532
9.0853
9.0777
9.0683
9.0606
8.1270
8.1194
8.0947
8.0870
8.0713
8.0412
7.9164
7.4465
7.4294
7.4144
7.3972
6.7785
6.6741
3.0873
3.0630
3.0335
2.8521
2.8219
2.7976
1.90
2.07
4.23
1.97
4.32
2.24
2.04
1.95
4.00
3.95
f1 (ppm)

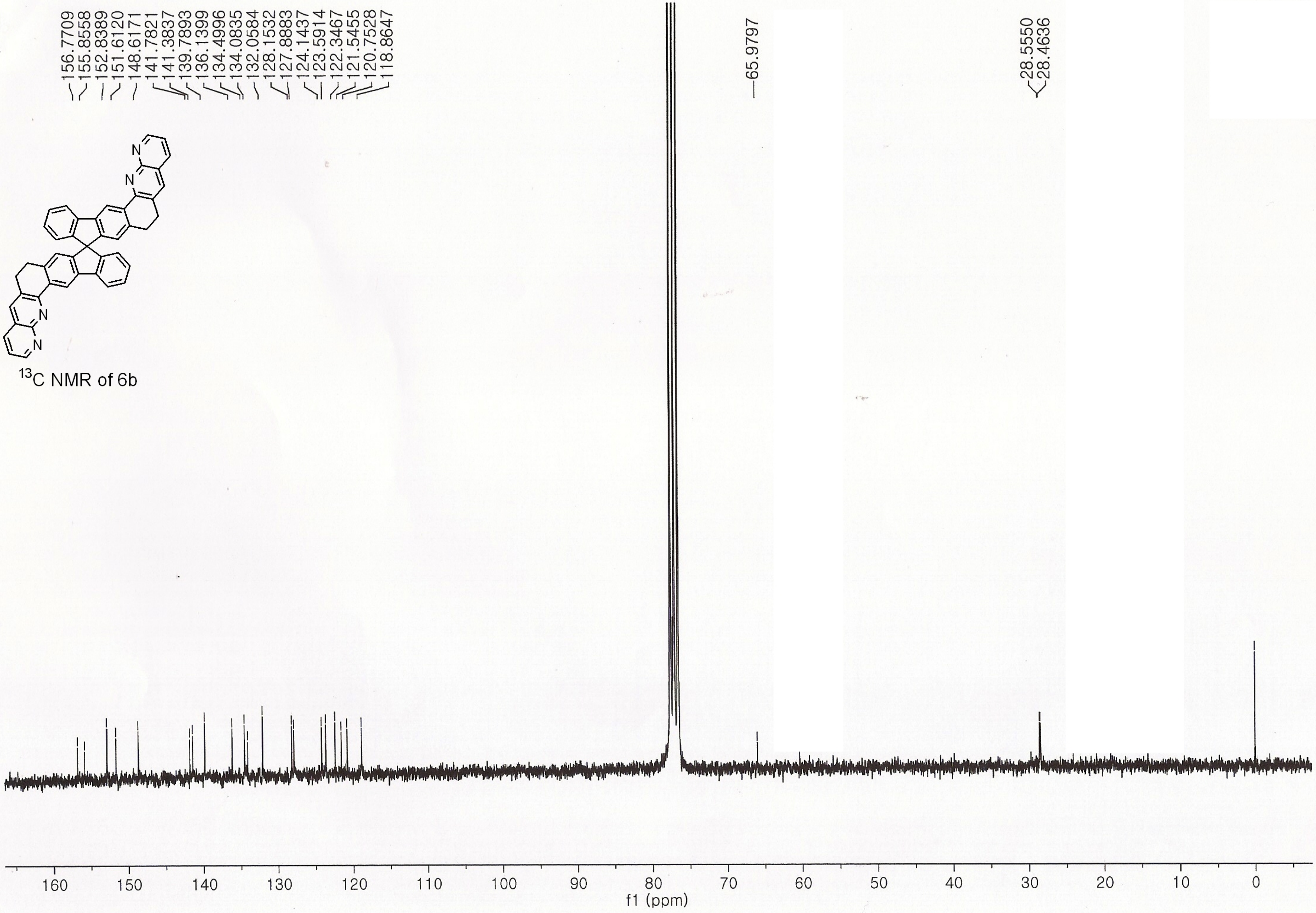

156.7709
155.8558
152.8389
151.6120
148.6171
141.7821
141.3837
139.7893
136.1399
134.4996
134.0835
132.0584
128.1532
127.8883
124.1437
123.5914
122.3467
121.5455
120.7528
118.8647
65.9797
28.5550
28.4636
160 150 140 130 120 110 100 90 80 70 60 50 40 30 20 10 0
f1 (ppm)

¹³C NMR of 6b

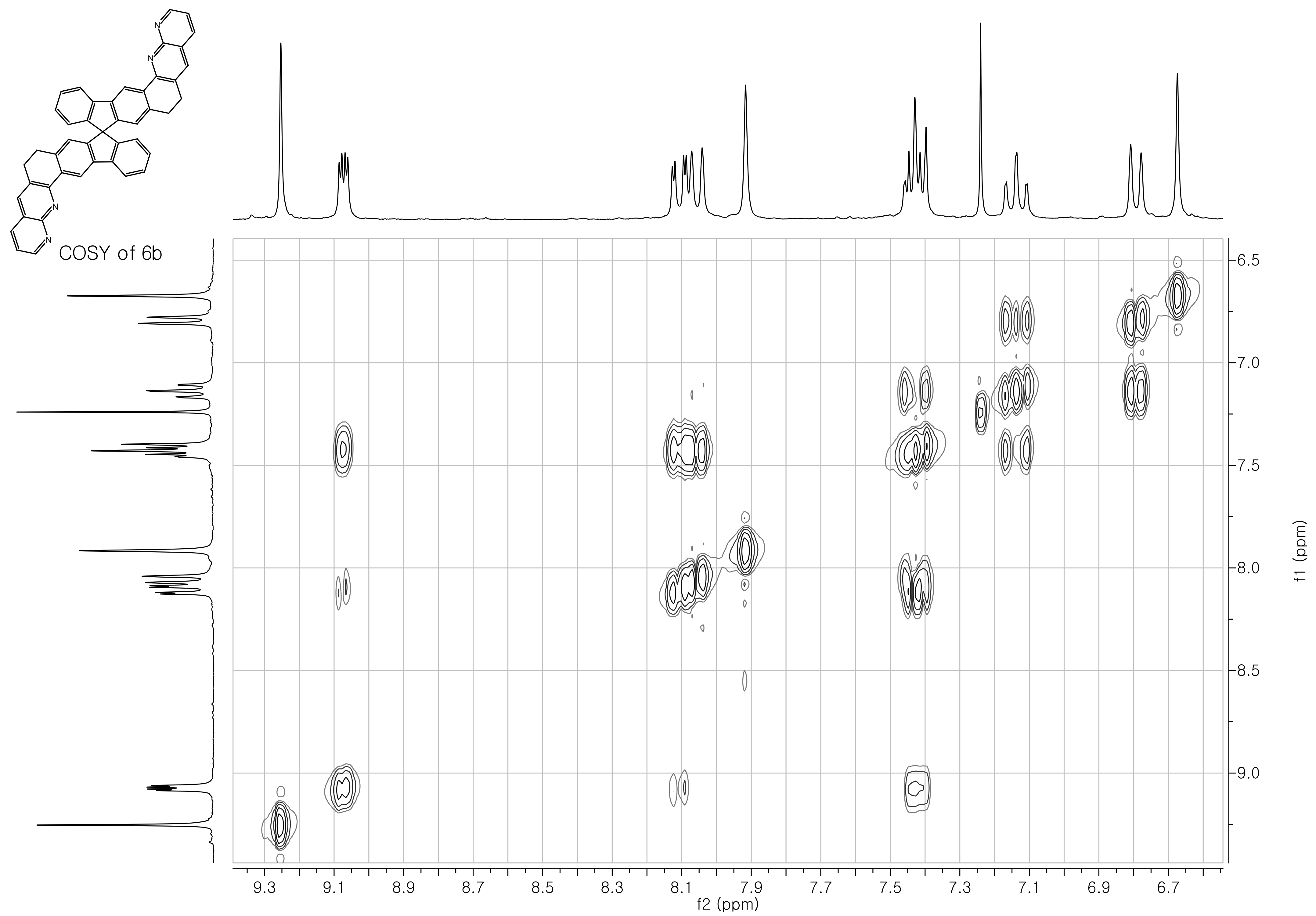

COSY of 6b
N
N
N
N
6.5
7.0
7.5
8.0
8.5
9.0
f1 (ppm)
9.3
9.1
8.9
8.7
8.5
8.3
8.1
7.9
7.7
7.5
7.3
7.1
6.9
6.7
f2 (ppm)

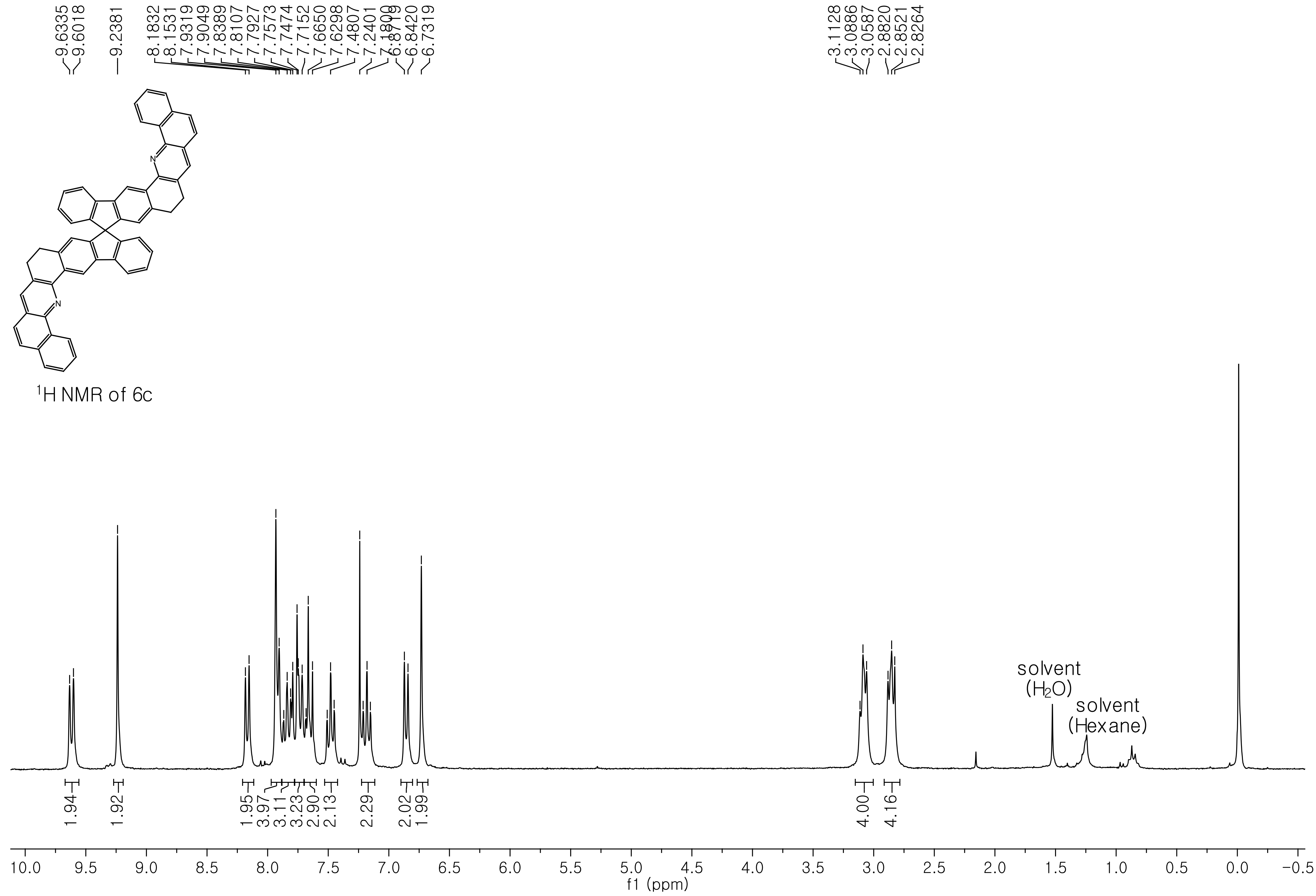

9.6335
9.6018
9.2381
8.1832
8.1531
7.9319
7.9049
7.8389
7.8107
7.7927
7.7573
7.7474
7.7152
7.6650
7.6298
7.4807
7.2401
7.1800
6.8719
6.8420
6.7319
3.1128
3.0886
3.0587
2.8820
2.8521
2.8264
N
N
¹H NMR of 6c
solvent
(H2O)
solvent
(Hexane)
1.94
1.92
1.95
3.97
3.11
3.23
2.90
2.13
2.29
2.02
1.99
4.00
4.16
10.0
9.5
9.0
8.5
8.0
7.5
7.0
6.5
6.0
5.5
5.0
4.5
4.0
3.5
3.0
2.5
2.0
1.5
1.0
0.5
0.0
−0.5
f1 (ppm)

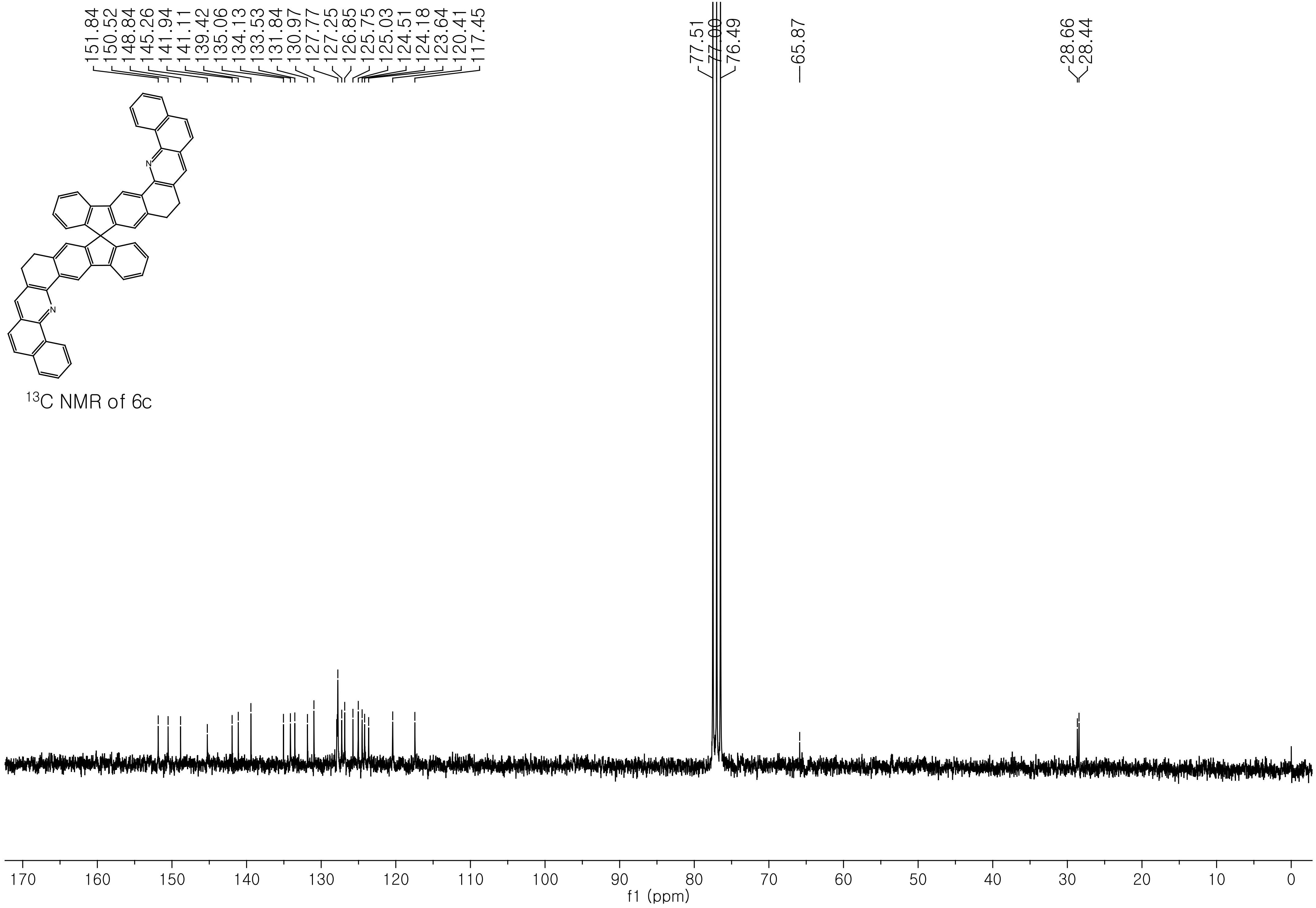
151.84
150.52
148.84
145.26
141.94
141.11
139.42
135.06
134.13
133.53
131.84
130.97
127.77
127.25
126.85
125.75
125.03
124.51
124.18
123.64
120.41
117.45
77.51
77.00
76.49
65.87
28.66
28.44
N
N
170
160
150
140
130
120
110
100
90
80
70
60
50
40
30
20
10
0
f1 (ppm)

^{13}C NMR of 6c

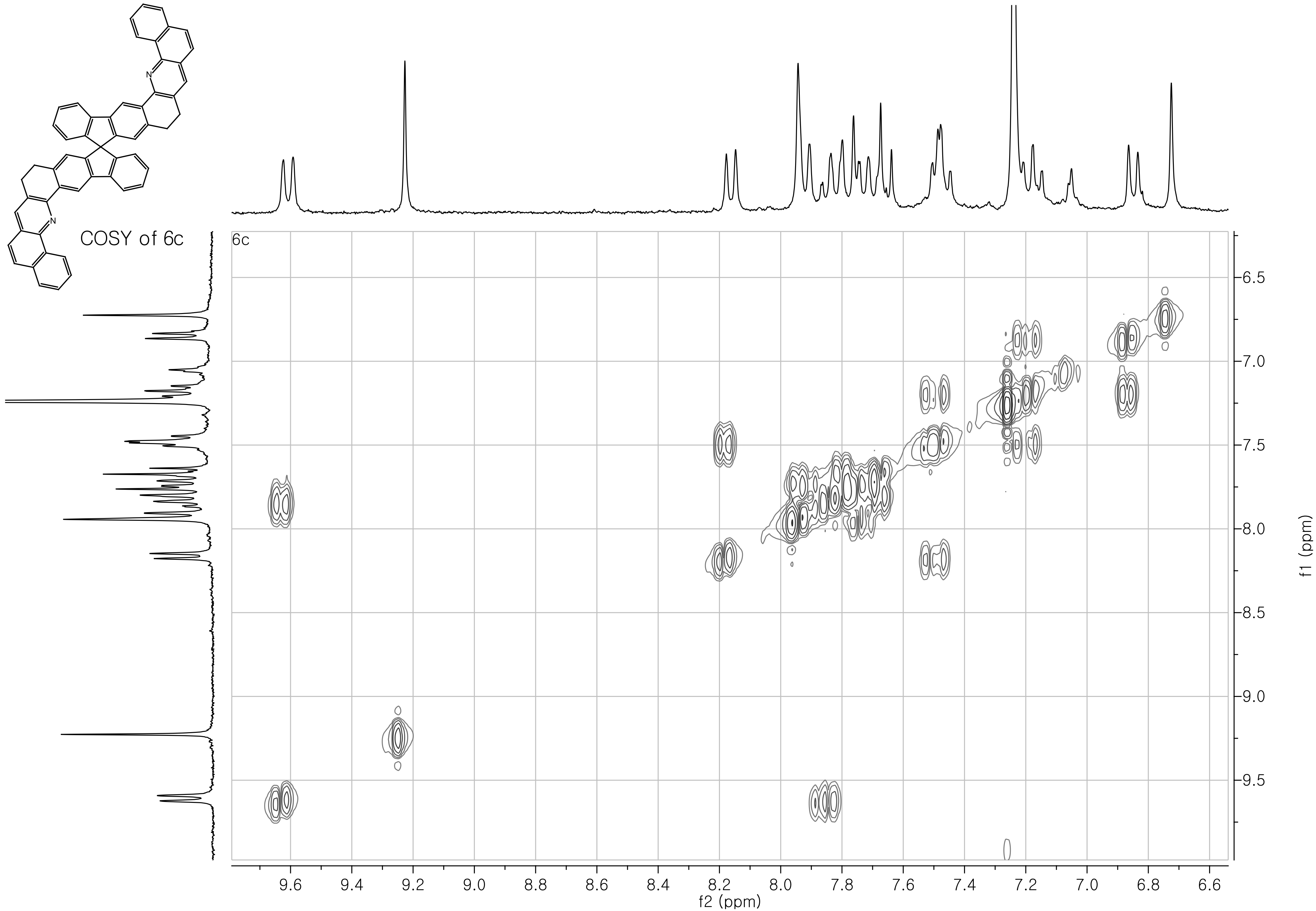

COSY of 6c

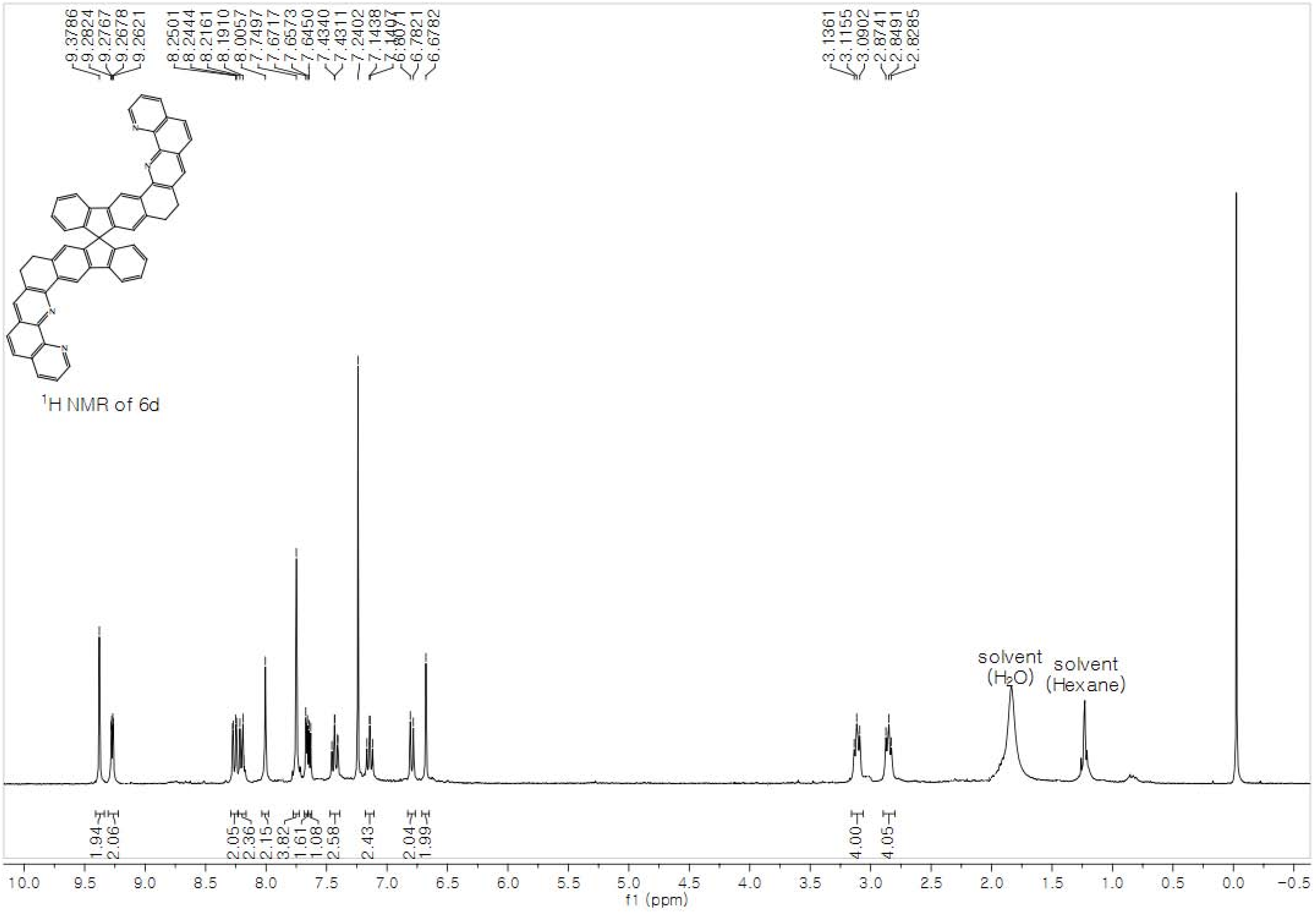
9.3786
9.2824
9.2767
9.2678
9.2621
8.2501
8.2444
8.2161
8.1910
8.0057
7.7497
7.6717
7.6573
7.6450
7.4340
7.4311
7.2402
7.1438
7.1407
6.8071
6.7821
6.6782
3.1361
3.1155
3.0902
2.8741
2.8491
2.8285
N
N
N
N
^{1}H NMR of 6d
solvent
(H_2O)
solvent
(Hexane)
1.94
2.06
2.05
2.36
2.15
3.82
1.61
1.08
2.58
2.43
2.04
1.99
4.00
4.05
10.0
9.5
9.0
8.5
8.0
7.5
7.0
6.5
6.0
5.5
5.0
4.5
4.0
3.5
3.0
2.5
2.0
1.5
1.0
0.5
0.0
-0.5
f1 (ppm)

¹³C NMR of 6d

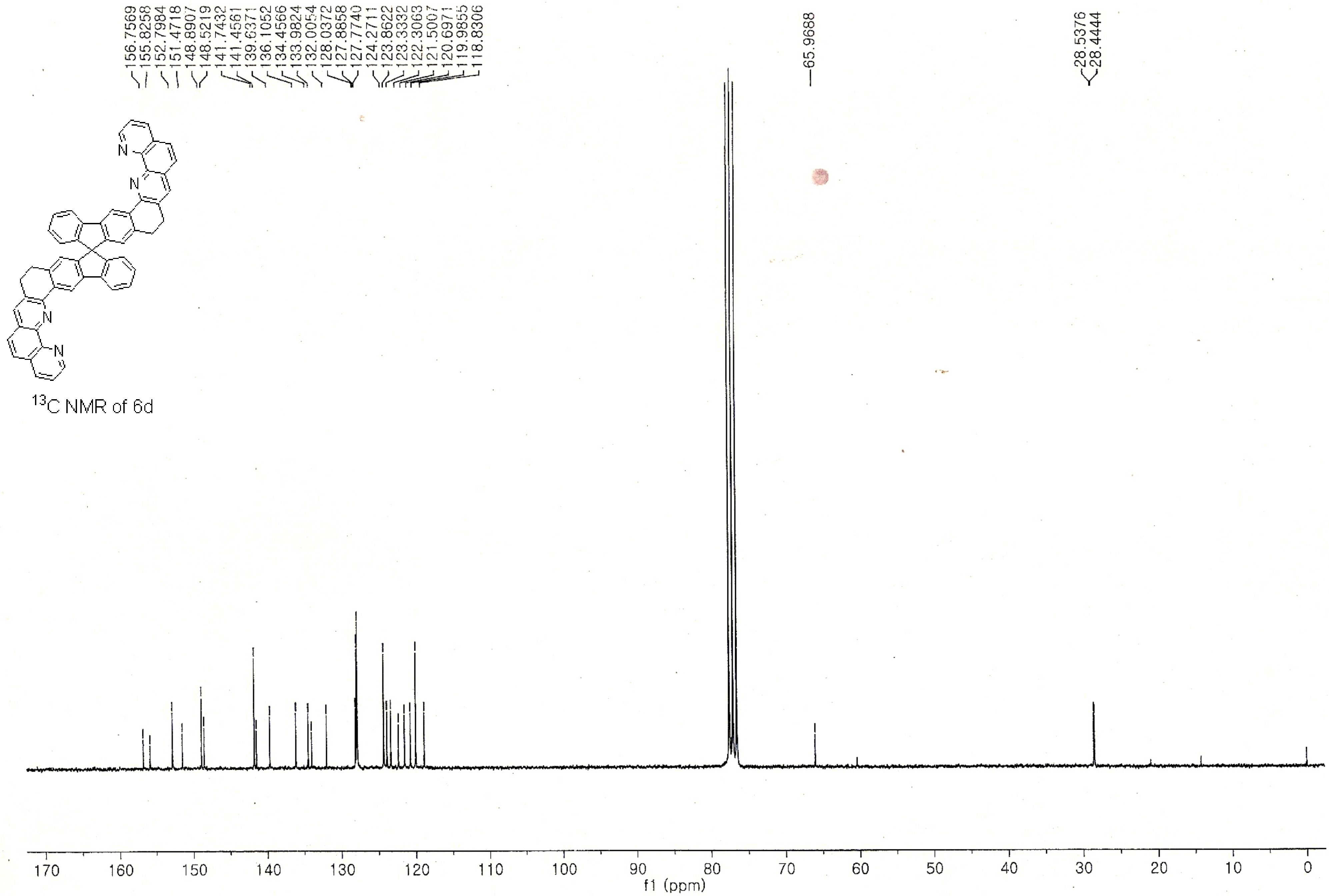
156.7569
155.8258
152.7984
151.4718
148.8907
148.5219
141.7432
141.4561
139.6371
136.1052
134.4566
133.9824
132.0054
128.0372
127.8858
127.7740
124.2711
123.8622
123.3332
122.3063
121.5007
120.6971
119.9855
118.8306
65.9688
28.5376
28.4444
170 160 150 140 130 120 110 100 90 80 70 60 50 40 30 20 10 0
f1 (ppm)

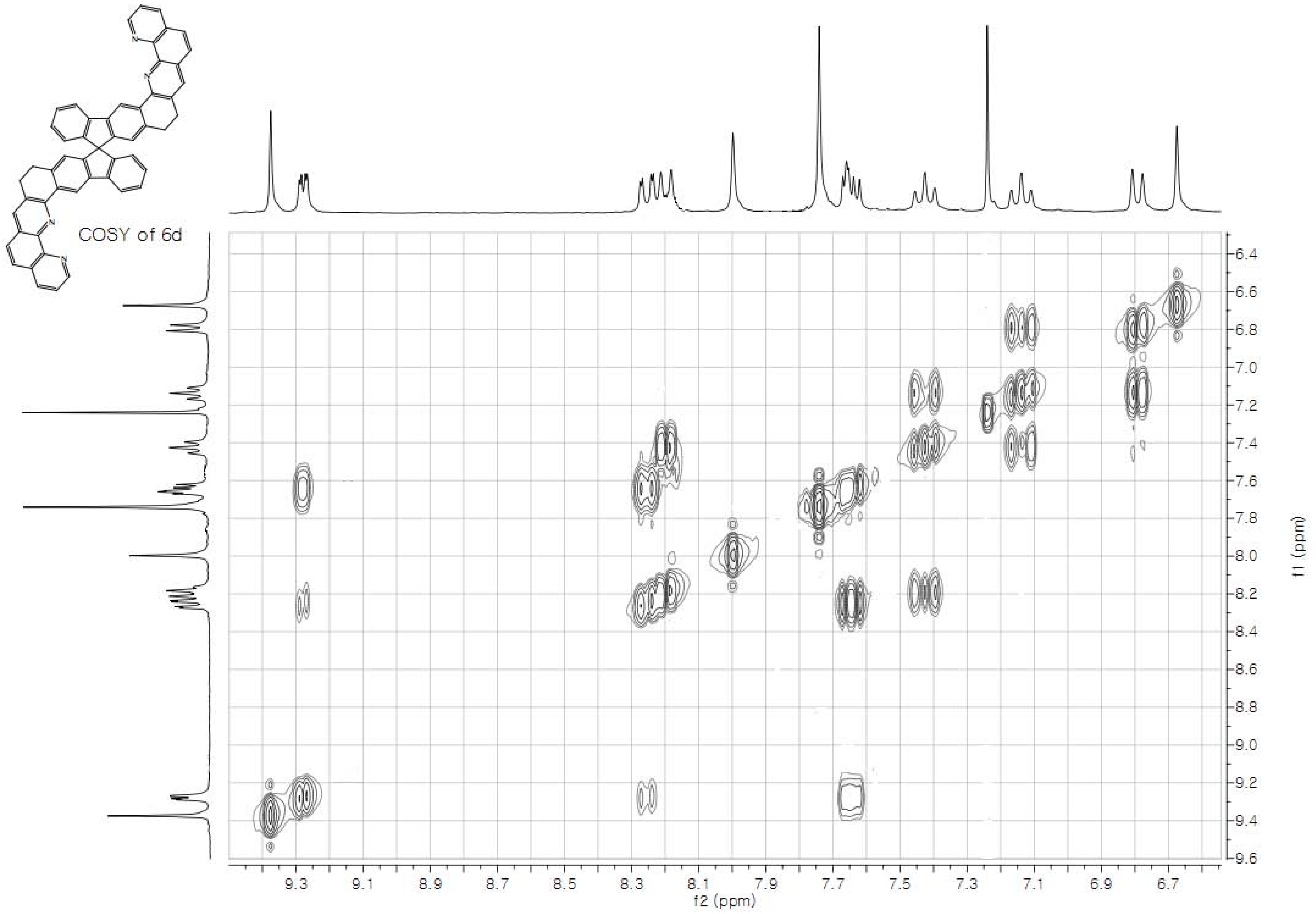
COSY of 6d
f2 (ppm)
f1 (ppm)
9.3
9.1
8.9
8.7
8.5
8.3
8.1
7.9
7.7
7.5
7.3
7.1
6.9
6.7
6.4
6.6
6.8
7.0
7.2
7.4
7.6
7.8
8.0
8.2
8.4
8.6
8.8
9.0
9.2
9.4
9.6
